# Deep Sequencing of B Cell Receptor Repertoires From COVID-19 Patients Reveals Strong Convergent Immune Signatures

**DOI:** 10.3389/fimmu.2020.605170

**Published:** 2020-12-15

**Authors:** Jacob D. Galson, Sebastian Schaetzle, Rachael J. M. Bashford-Rogers, Matthew I. J. Raybould, Aleksandr Kovaltsuk, Gavin J. Kilpatrick, Ralph Minter, Donna K. Finch, Jorge Dias, Louisa K. James, Gavin Thomas, Wing-Yiu Jason Lee, Jason Betley, Olivia Cavlan, Alex Leech, Charlotte M. Deane, Joan Seoane, Carlos Caldas, Daniel J. Pennington, Paul Pfeffer, Jane Osbourn

**Affiliations:** ^1^ Alchemab Therapeutics Ltd, London, United Kingdom; ^2^ Wellcome Centre for Human Genetics, Oxford, United Kingdom; ^3^ Oxford Protein Informatics Group, Department of Statistics, University of Oxford, Oxford, United Kingdom; ^4^ Barts and The London School of Medicine and Dentistry, Queen Mary University of London, London, United Kingdom; ^5^ Illumina, Inc., Illumina Centre, Cambridge, United Kingdom; ^6^ Translational Research Program, Vall d’Hebron Institute of Oncology, Barcelona, Spain; ^7^ Cancer Research UK Cambridge Institute and Department of Oncology, Li Ka Shing Centre, University of Cambridge, Cambridge, United Kingdom

**Keywords:** COVID-19, SARS-CoV-2, B-cell repertoire, BCR, antibody, convergence

## Abstract

Deep sequencing of B cell receptor (BCR) heavy chains from a cohort of 31 COVID-19 patients from the UK reveals a stereotypical naive immune response to SARS-CoV-2 which is consistent across patients. Clonal expansion of the B cell population is also observed and may be the result of memory bystander effects. There was a strong convergent sequence signature across patients, and we identified 1,254 clonotypes convergent between at least four of the COVID-19 patients, but not present in healthy controls or individuals following seasonal influenza vaccination. A subset of the convergent clonotypes were homologous to known SARS and SARS-CoV-2 spike protein neutralizing antibodies. Convergence was also demonstrated across wide geographies by comparison of data sets between patients from UK, USA, and China, further validating the disease association and consistency of the stereotypical immune response even at the sequence level. These convergent clonotypes provide a resource to identify potential therapeutic and prophylactic antibodies and demonstrate the potential of BCR profiling as a tool to help understand patient responses.

## Introduction

Since the report of the first patients in December 2019 (1, 2), the unprecedented global scale of the COVID-19 pandemic has become apparent. The infectious agent, the SARS-CoV-2 betacoronavirus (3), causes mild symptoms in most cases but can cause severe respiratory diseases such as acute respiratory distress syndrome in some individuals. Risk factors for severe disease include age, male gender, and underlying co-morbidities (4).

Understanding the immune response to SARS-CoV-2 infection is critical to support the development of therapies. Recombinant monoclonal antibodies derived from analyses of B cell receptor (BCR) repertoires in infected patients or the immunization of animals have been shown to be effective against several infectious diseases including Ebola virus (5), rabies (6), and respiratory syncytial virus disease (7). Such therapeutic antibodies have the potential to protect susceptible populations as well as to treat severe established infections.

While many vaccine approaches are underway in response to the SARS-CoV-2 outbreak, many of these compositions include as immunogens either whole, attenuated virus or whole spike (S) protein—a viral membrane glycoprotein which mediates cell uptake by binding to host angiotensin-converting enzyme 2 (ACE2). The antibody response to such vaccines will be polyclonal in nature and will likely include both neutralizing and non-neutralizing antibodies. It is hoped that the neutralizing component will be sufficient to provide long-term SARS-CoV-2 immunity following vaccination, although other potential confounders may exist, such as raising antibodies which mediate antibody-dependent enhancement (ADE) of viral entry (8–10). While ADE is not proven for SARS-CoV-2, prior studies of SARS-CoV-1 in non-human primates showed that, while some S protein antibodies from human SARS-CoV-1 patients were protective, others enhanced the infection *via* ADE (11). An alternative could be to support passive immunity to SARS-CoV-2, by administering one, or a small cocktail of, well-characterized, neutralizing antibodies.

Patients recovering from COVID-19 have already been screened to identify neutralizing antibodies, following analysis of relatively small numbers (100–500) of antibody sequences (12–14). A more extensive BCR repertoire analysis was performed on six patients in Stanford, USA with signs and symptoms of COVID-19 who also tested positive for SARS-CoV-2 RNA (15). Although no information was provided on the patient outcomes in that study, the analysis demonstrated preferential expression of a subset of immunoglobulin heavy chain (IGH) V gene segments with relatively little somatic hypermutation and showed evidence of convergent antibodies between patients.

To drive a deeper understanding of the nature of humoral immunity to SARS-CoV-2 infection and to identify potential therapeutic antibodies to SARS-CoV-2, we have evaluated the BCR heavy chain repertoire from 31 individuals at various stages of their immune response. We show that there are stereotypic responses to SARS-CoV-2 infection, that infection stimulates both naïve and memory B cell responses, that sequence convergence can be used to identify putative SARS-CoV-2 specific antibodies, and that sequence convergence can be identified between different SARS-CoV-2 studies in different locations and using different sample types.

## Methods

### Clinical Information Gathering

Peripheral blood was obtained from patients admitted with acute COVID-19 pneumonia to medical wards at Barts Health NHS Trust, London, UK, after informed consent by the direct care team (NHS HRA RES Ethics 19/SC/0361). Venous blood was collected in 20-ml EDTA Vacutainers (BD). Patient demographics and clinical information relevant to their admission were collected by members of the direct care team, including duration of symptoms prior to blood sample collection. Current severity was mapped to the WHO Ordinal Scale of Severity. Whether patients at time of sample collection were clinically Improving, Stable or Deteriorating was subjectively determined by the direct clinical team prior to any sample analysis. This determination was primarily made on the basis of whether requirement for supplemental oxygen was increasing, stable, or decreasing comparing current day to previous 3 days. Lymphocyte counts were determined using a standard clinical cytometer.

### Sample Collection and Initial Processing

Blood samples were processed within 1 h of collection in order to limit RNA degradation. Blood was first centrifuged at 150 x*g* for 15 min at room temperature to separate plasma. The cell pellet was then resuspended with phosphate-buffered saline (PBS without calcium and magnesium, Sigma) to 20 ml, layered onto 15-ml Ficoll-Paque Plus (GE Healthcare) and then centrifuged at 400 x*g* for 30 min at room temperature without brake. Mononuclear cells (PBMCs) were extracted from the buffy coat and washed twice with PBS at 300 x*g* for 8 min. PBMCs were counted with Trypan blue (Sigma) and viability of >96% was observed. PBMCs (5 × 10^6^) were immediately resuspended in RLT buffer to stabilize the RNA (Qiagen) and incubated at room temperature for 10 min prior to storage at –80°C. Consecutive donor samples with sufficient RLT samples progressed to RNA preparation and BCR preparation and are included in this manuscript.

Metastatic breast cancer biopsy samples were collected and RNA extracted as part of a previously reported cohort (16).

### RNA Prep and BCR Sequencing

Total RNA from 5 × 10^6^ PBMCs was isolated using RNeasy kits (Qiagen). First-strand cDNA was generated from total RNA using SuperScript RT IV (Invitrogen) and IgA and IgG isotype specific primers (17) including UMIs at 50°C for 45 min (inactivation at 80°C for 10 min).

The resulting cDNA was used as template for High Fidelity PCR amplification (KAPA, Roche) using a set of 6 FR1-specific forward primers (17) including sample-specific barcode sequences (6 bp) and a reverse primer specific to the RT primer (initial denaturation at 95°C for 3 min, 25 cycles at 98°C for 20 s, 60°C for 30 s, 72°C for 1 min and final extension at 72°C for 7 min). The amount of BCR heavy chain amplicons (~450 bp) was quantified by TapeStation (Beckman Coulter) and gel-purified.

Dual-indexed sequencing adapters (KAPA) were ligated onto 500-ng amplicons per patient using the HyperPrep library construction kit (KAPA) and the adapter-ligated libraries were finally PCR-amplified for 3 cycles (98°C for 15 s, 60°C for 30 s, 72°C for 30s, final extension at 72°C for 1 min). Pools of 10, 9, and 12 libraries were sequenced across three runs on an Illumina MiSeq using 2 × 300 bp chemistry.

### Sequence Processing

The Immcantation framework (docker container v3.0.0) was used for sequence processing (18, 19). Briefly, paired-end reads were joined based on a minimum overlap of 20 nt, and a max error of 0.2, and reads with a mean phred score below 20 were removed. Primer regions, including UMIs and sample barcodes, were then identified within each read, and trimmed. Together, the sample barcode, UMI, and constant region primer were used to assign molecular groupings for each read. Within each grouping, usearch (20) was used to subdivide the grouping, with a cutoff of 80% nucleotide identity, to account for randomly overlapping UMIs. Each of the resulting groupings is assumed to represent reads arising from a single RNA. Reads within each grouping were then aligned, and a consensus sequence determined. Finally, duplicate reads were collapsed into a single processed sequence for analysis. Collapsing duplicate reads ensures that each processed sequence represents a sequence from a single B cell and our analysis is not confounded by expression level.

For each processed sequence, IgBlast (21) was used to determine V, D and J gene segments, and locations of the complementarity determining regions (CDRs) and framework regions (FWRs). Isotype was determined based on comparison to germline constant region sequences. Sequences annotated as unproductive by IgBlast were removed. The number of mutations within each sequence was determined using the shazam R package (19).

### Public BCR Sequence Data Processing

The healthy control BCR sequence dataset used here has been described previously (22). It was prepared using the same primer set that was used in the current study, so comparisons should not suffer from protocol-specific biases. Only samples from participants aged 10 years or older, and from peripheral blood were used. This resulted in samples from 40 different participants, with a mean age of 28 (range: 11–51). Furthermore, only class-switched sequences were considered.

The influenza vaccine BCR sequence dataset used here was a combination of data from two different studies, and amounted to samples from 6 different participants in total (23, 24). All participants were administered a seasonal influenza vaccine, and peripheral blood was taken for BCR sequencing 6–9 days following vaccination. The processed data from these studies was obtained directly from the Observed Antibody Space database, and only the class-switched sequences were considered (25).

The publicly available BCR sequence dataset from six COVID-19 patients in Stanford, USA was also obtained directly from the Observed Antibody Space database. To circumvent the disparity in collapsed dataset sizes between pairs of replicates, we selected the replicate with the highest number of sequences for downstream analysis.

### Clonotyping

BCR sequences were clustered to identify those arising from presumably clonally related B cells; a process termed clonotyping. Sequences from the 31 COVID-19 patients, the 7 metastatic cancer patients, the 6 influenza vaccine participants, and the 40 healthy controls were clustered together to also identify convergent clusters between samples. Clustering was performed using a previously described algorithm (26). Clustering required identical V and J gene segment usage, identical CDRH3 length, and allowed 1 AA mismatch for every 10 AAs within the CDRH3. Cluster centers were defined as the most common sequence within the cluster. Lineages were reconstructed from clusters using the alakazam R package (27).

### Bronchoalveolar Lavage RNAseq Data Processing

The bronchoalveolar lavage data comes from a previously published study of SARS-CoV-2 infection (28), with data available under the PRJNA605983 BioProject on NCBI. MIXCR v3.0.3 was used, with default settings, to extract reads mapping to antibody genes from the total RNASeq data (29).

### Convergent Clonotype Matching to Public Data

The public COVID-19 BCR sequence dataset (15), the output from the SARS-CoV-2 RNAseq data processing (28), and sequences from CoV-AbDab (30) were scanned for matches to our 1,254 convergent clonotype cluster centers. For comparison to the COVID-19 BCR sequence dataset and the RNAseq dataset, matching was performed using the same threshold that was used for clonotyping – requiring identical V and J genes, the same length CDRH3, and within 1 AA mismatch per 10 CDRH3 AAs to the convergent clonotype cluster center. For matching to the CoV-AbDab, a more lenient threshold was used. Sequences from CoV-AbDab were clustered alongside the representative CDRH3 sequence from each of our 1,254 convergent clones using the cd-hit-2d script from the CD-HIT package (31), at an 80% sequence identity threshold. Clustered CDRH3s were not required to have the same length, however the shorter CDRH3 was required to be at least 90% of the length of the longer CDRH3. Cluster centers containing at least one CoV-AbDab CDRH3 and one convergent clonotype CDRH3 were further investigated.

### Statistical Analysis and Graphing

Statistical analysis and plotting were performed using R (32). Plotting was performed using ggplot2 (33). Specific statistical tests used are detailed in the figure legends. Correlations of IGHV4-34 autoreactive motifs was performed by MANOVA in R.

## Results

### COVID-19 Disease Samples

Blood samples were collected from thirty-one patients admitted to hospital with acute COVID-19 pneumonia. The mean age of patients was 56.2 (SD 17.7) years and 20 (65%) were male (**Supplementary Table 1**). All patients had a clinical history consistent with COVID-19 and typical radiological changes. Twenty-eight patients had a confirmatory positive PCR test for SARS-CoV-2. The patients experienced an average of 16 days (range 4–55) of symptoms prior to the day on which the blood sample was collected. Twelve of the patients were still requiring hospital care but not oxygen therapy on day of sample collection (WHO Ordinal Scale Score 3), while 13 were hospitalized requiring oxygen by conventional mask or nasal prongs (WHO Ordinal Scale Score 4) and six were hospitalized with severe COVID-19 pneumonia requiring high-flow nasal oxygen or continuous positive airway pressure by mask (WHO Ordinal Scale Score 5). On the day of sample collection, the direct clinical care team considered two patients to be deteriorating, ten improving and the remaining nineteen were clinically stable.

It has previously been observed that lymphopenia is seen in severe COVID-19 patients (34). Our results recapitulate these findings, showing a correlation between lymphocyte count and clinical status (**Supplementary Figure 1**). As COVID-19 lymphopenia is caused by a reduction in T cell rather than B cell counts, we do not expect the different lymphocyte counts from our patients to impact the BCR sequencing results.

### SARS-CoV-2 Infection Results in a Stereotypic B Cell Response

**Figure 1:**
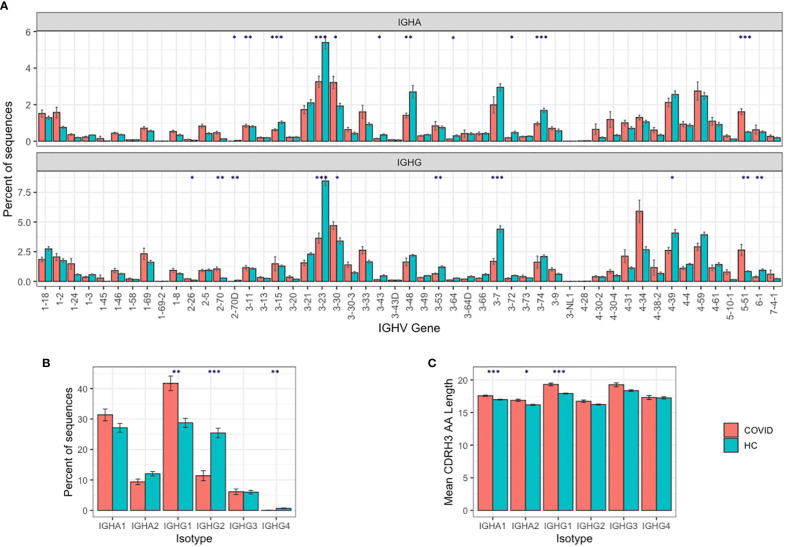
B cell responses to SARS-COV-2 infection. **(A)** IGHV gene segment usage distribution per isotype. **(B)** Isotype subclass distribution between IGHA and IGHG subclasses, and **(C)** mean BCR CDRH3 lengths from COVID-19 patients compared to healthy controls. For **(A–C)**, bars show mean values +/− standard error of the mean. Comparisons performed using t-tests, with adjusted p values using Bonferroni correction for multiple comparisons; * p < 0.05, ** p < 0.005, *** p < 0.0005.

IGHA and IGHG BCR sequencing yielded on average 154,717 unique sequences, and 24,043 clonotypes per sample (**Supplementary Table 1**). To characterize the B cell response in COVID-19, we compared this BCR repertoire data to BCR repertoire data from healthy controls obtained in a separate study (**Supplementary Table 2**), but amplified using the same primer set (22). Comparing IGHV gene segment usage revealed a significantly different IGHV gene usage in COVID-19 patients compared to the healthy controls, most notably with increases in the usage of IGHV2-70 (3.6× IGHA, 3.9× IGHG increase), IGHV3-30 (1.7× IGHA, 1.4× IGHG increase), IGHV5-51 (3.2× IGHA, 3.2× IGHG increase), and IGHV4-34 (1.2× IGHA, 2.2× IGHG increase) in the COVID-19 patients (**Figure 1A**).

We further investigated the IGHV4-34 difference, as IGHV4-34 has been shown to produce self-reactive antibodies (35). The IGHV4-34 germline sequence contains two sequence motifs (an “AVY” hydrophobic patch in the FWR1, and an “NHS” glycosylation sequon in the CDR2) that are not found in other IGHV gene segments and are thought to drive the self-reactivity. We show that the proportion of sequences containing the autoreactive AVY & NHS sequence motifs is increased in improving COVID-19 patients compared to stable or deteriorating COVID-19 patients, specifically in the IGHG1 isotype subclass (p-value = 0.013; **Supplementary Figure 2**).

Comparing isotype subclasses showed a significant increase in the relative usage of IGHG1 in COVID-19 patients (**Figure 1B**)—this is the first isotype subclass that is switched to upon activation of IGHM (36). There was also an increase in the mean CDRH3 length of the BCRs in the COVID-19 patients, that was most pronounced in the IGHA1, IGHA2, and IGHG1 isotype subclasses (**Figure 1C**).

### SARS-CoV-2 Infection Stimulates Both Naïve and Memory Responses

**Figure 2:**
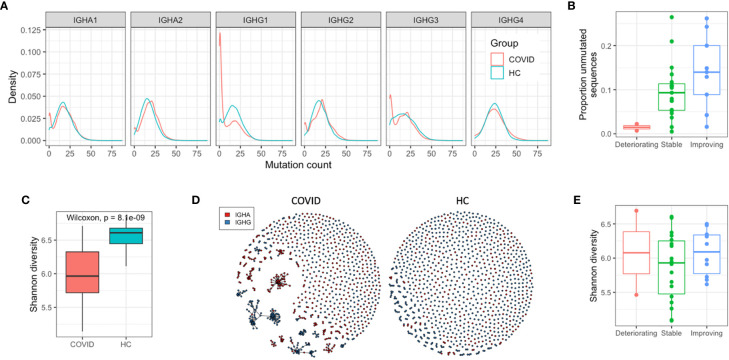
Response characteristics of SARS-CoV-2 infection. **(A)** Distribution of sequences with different numbers of mutations from germline. **(B)** Relationship between the proportion of the repertoire comprised by unmutated sequences, and the disease state **(C)** Individual sequences were clustered together into related groups to identify clonal expansions (clonotypes). Diversity of all clonotypes in the repertoire calculated using the Shannon diversity index. To normalize for different sequence numbers for each sample, a random subsample of 1,000 sequences was taken. **(D)** Network graphs of a representative repertoire from a single COVID-19 patient, and a single HC sample giving a graphical representation of the diversity. Each point represents one of the subsampled sequences, and sequences within the same clonotype are linked together. **(E)** Relationship between repertoire diversity and disease state.

To further investigate the COVID-19-specific B cell response, we analyzed the characteristics of the BCR sequences that are consistent with recent B cell activation—somatic hypermutation and clonal expansion. In healthy controls, for class-switched sequences, there is a clear unimodal distribution of sequences with different numbers of mutations, and a mean mutation count across IGHA and IGHG isotypes of 17.6 (**Figure 2A**). In the COVID-19 samples, the mean mutation count was 14.8, and there was a bimodal distribution with a separate peak of sequences with no mutations. This bimodal distribution was most pronounced in the IGHG1, IGHG3, and IGHA1 isotype subclasses, corresponding to the increased isotype usages. Such a distribution is consistent with an expansion of recently class-switched B cells that have yet to undergo somatic hypermutation. There was considerable variation between participants in the proportion of unmutated sequences (**Supplementary Figure 3**), which had no significant correlation with the number of days since symptom onset (R = −0.098, p = 0.61), but was lower in the deteriorating compared to improving patients (**Figure 2B**).

To investigate the clonal expansion in these antigen experienced BCR repertoires in more detail, the Shannon diversity index of each repertoire was calculated (while accounting for differences in read depth through subsampling). Lower diversity is indicative of a small number of dominant clonal expansions, while higher diversity is indicative or a larger number of smaller clonal expansions. The class-switched BCR repertoires of the COVID-19 patients were significantly less diverse than the BCR repertoires of the healthy controls due to the presence of large clonal expansions (**Figures 2C, D**), but there was no relationship between disease state and BCR repertoire diversity (**Figure 2E**). Calculating the mean mutation count of the clonal expansions showed that the largest ones were all highly mutated; the mean mutation of the ten largest clonal expansions in each COVID-19 patient repertoire was 18.4. Taken together, these observations suggest the response to COVID-19 consists of both recently activated B cells with low levels of mutation, and historic memory B cells that have high levels of mutation, and form particularly large clonal expansions.

### Sequence Convergence Can Be Used to Identify Putative SARS-CoV-2 Specific Antibodies

Given the skewing of the B cell response in the COVID-19 patients to specific IGHV genes, we next investigated whether the same similarity was also seen on the BCR sequence level between different participants. Such convergent BCR signatures have been observed in response to other infectious diseases (37) and may be used to identify disease-specific antibody sequences.

**Figure 3:**
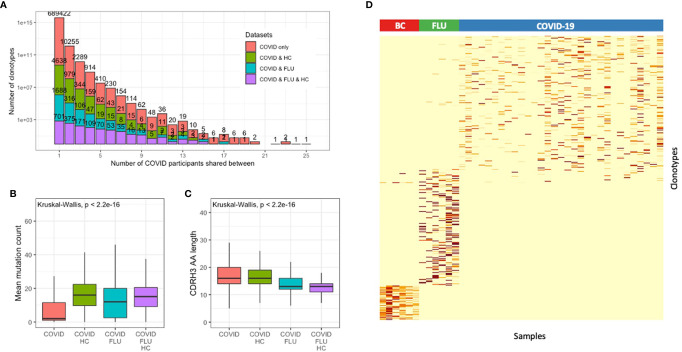
Convergent BCR sequence signature within individuals infected with SARS-CoV-2. **(A)** Data from all patients and healthy controls were clustered together to identify convergent clonotypes. Shown is the number of clonotypes shared by different numbers of participants, grouped by whether the clonotypes are also present in the healthy control dataset. Of the convergent clonotypes, **(B)** the mean mutation count, and **(C)** the CDRH3 AA sequence length was compared between those that were convergent only within the SARS-CoV-2 patients, and those that were also convergent with the healthy control dataset. **(D)** Heatmap of the 1,254 convergent COVID-19-associated clonotypes (observed between 4 or more COVID-19 participants) with the 1,180 convergent clonotypes from six influenza vaccination (FLU) samples, and 351 convergent clonotypes from six metastatic breast cancer (BC) patient biopsy samples, demonstrating that the convergent signatures are unique to each cohort.

Of the 714,107 total clonotypes across all the COVID-19 patients, 17,658 (2.5%) were shared between at least two of the participants (**Figure 3A**). Sample-to-sample contamination within sequencing batches can erroneously be interpreted as convergence, so we used a dual sample barcoding method, and strict laboratory procedures to minimize this. Although an element of contamination can never entirely be ruled out, we found no batch effects between the three sequencing runs we conducted (**Supplementary Figure 4**). As convergence could also occur by chance or be due to an unrelated memory response from commonly encountered pathogens, additional datasets were incorporated into the convergence analysis. The healthy control dataset was used to identify BCR sequences that may be shared by chance or come from commonly encountered pathogens. In addition, a BCR sequence dataset of six individuals who had received an influenza vaccination 6–9 days prior to sampling, was used to further identify BCR sequences that could arise during general respiratory tract infection (23, 24).

Of the 17,658 convergent clonotypes, 2,530 (14.3%) were also present in at least one of the 40 healthy control samples, 1,400 (7.9%) were also present in at least one of the 6 influenza samples, and 869 (4.9%) were also present in at least one of the healthy controls and one of the influenza samples. As expected, of the convergent clonotypes that were also present in the healthy control or influenza samples, the mean mutation count was significantly greater (**Figure 3B**), and the mean CDRH3 length significantly shorter (**Figure 3C**) than the convergent clonotypes that were unique to the COVID-19 patients.

To identify a set of potentially SARS-CoV-2-specific antibody sequences with high confidence, we first selected convergent clonotypes that were shared between at least four of the COVID-19 patients, but not seen in the healthy controls. This revealed 1,337 total clonotypes. For comparison, the same analysis was also conducted on the influenza sample dataset but using a more lenient threshold of sharing between at least two samples in order to identify a similar total number of 1,180 total clonotypes. Finally, as a technical control to determine whether the convergent signature could represent laboratory contamination, the same analysis was performed on an unrelated set of six metastatic breast cancer patient biopsy samples (16), which identified 351 convergent clonotypes. These convergent clonotypes identified from each of the COVID-19, influenza and metastatic cancer cohorts were highly specific to each sample cohort (**Figure 3D**). Removing the small amount of overlap that the 1,337 COVID-19 convergent clonotypes had with the influenza and metastatic cancer samples resulted in a final signature of 1,254 convergent clonotypes.

The 1,254 COVID-19 convergent clonotypes had low mutation levels, with a mean mutation count of 2.3, and only 115 clonotypes with a mean mutation greater than 5. The sequences within the convergent clonotypes were primarily of the IGHG1 (71%) and IGHA1 (15%) subclasses (**Supplementary Figure 5A**). The convergent clonotypes used a diversity of IGHV gene segments, with IGHV3-30, IGHV3-30-3 and IGHV3-33 as the most highly represented (**Supplementary Figure 5B**). This IGHV gene usage distribution differs between that of the total repertoire, and IGHV3-30 is also the most highly used IGHV gene in the CoV-AbDab (30). The proportion of the repertoire comprised by each of these convergent clonotypes was also calculated for each patient sample. This proportion significantly correlated with decreased disease severity for one of the convergent clonotypes (IGHV3-30, CARGPGDSIDYW, IGHJ4) even after Bonferroni correction for multiple testing (p = 0.0052).

### BCR Clonotype Sequence Convergence Signatures are Shared Between Different COVID-19 Studies in Different Locations and From Different Anatomical Sites

To further explore whether the convergent clonotypes observed in our study were indeed disease specific, and to determine whether such convergence was common across studies and geographic regions, we compared these 1,254 convergent clonotypes to public B cell datasets. In addition, to ensure any matches to public datasets were not due to chance, we also selected at random 1,254 clonotypes from the healthy control set and performed the same comparison in parallel.

First, we compared our data to RNAseq data of bronchoalveolar lavage fluid obtained from five of the first infected patients in Wuhan, China (28). These samples were obtained for the purpose of metagenomic analyses to identify the aetiological agent of the novel coronavirus but were re-analyzed to determine whether we could extract any transcripts from BCRs. From the 10,038,758 total reads, we were able to identify 16 unique CDR3 AA sequences (**Supplementary Table 2**). Of these, one had an exact AA match to a CDR3 sequence in our data (CTTDLHDYGDSDYW) and shared the same V gene segment (IGHV3-15), and J gene segment (IGHJ4) usage. The clonotype that the sequence belonged to contained 796 total sequences and was convergent between 10 of our 31 COVID-19 patients. The clonotype was highly diverse, and the sequences had evidence of low mutation from germline, with a mean mutation count over all sequences of 5.3 (**Supplementary Figure 6**). None of the 16 CDR3 sequences from the RNAseq data matched to any of the CDR3 sequences in the 1,254 healthy control clonotypes, or indeed from any of the clonotypes in the entire dataset of 40 healthy control samples, indicating that the observed matching in the COVID-19 patients is unlikely just due to chance alone.

Next, we compared our 1,254 convergent clonotypes to CoV-AbDab – the Coronavirus Antibody Database (accessed 5^th^ August 2020) (30). At the time of access, this database contained 1,396 redundant and 1,151 non-redundant CDRH3 sequences from published and patented antibodies proven to bind coronaviruses including SARS-CoV-1 and SARS-CoV-2. We found 39 of our 1,254 convergent clonotypes to have high CDRH3 homology to the antibodies in CoV-AbDab (**Table 1** and **Supplementary Table 4**). In contrast, only one of the 1,254 healthy control clonotypes had high CDR3 homology to an antibody in CoV-AbDab. Of the 39 convergent clonotype matches, 10 also shared the same IGHV and IGHJ gene segment usage and would be classed as belonging to the same clonotype (**Table 1**); the healthy control clonotype match did not share the same IGHV or IGHJ gene segment usage.

**Table 1:** Convergent COVID-19 clonotypes with high CDRH3 homology to antibodies in the CoV-AbDab, and which share the same IGHV and IGHJ gene usage and CDRH3 length.

Name	CDRH3	IGHV	IGHJ	Binds	Neutralizes	Reference
C154	AKQAGPYCSGGSCYSAPFDY	3-30	4	CoV-1, CoV-2	CoV-2	Robbiani et al. (14)
*ALC_3983948*	**AKVSGPYCSGGSCYSFYFDY**	**3-30**	**4**			
COV2-2068	ARSYDILTGYRDAFDI	3-53	3	CoV-2	CoV-2	Zost et al. (38)
*ALC_2318471*	**VRNYDILTGYSDAFDI**	**3-53**	**3**			
COV2-2007	AKVSATYYYYYYGMDV	3-30	6	CoV-1, CoV-2		Zost et al. (38)
CC12.17	AKSSGSYYYYYYGMDV	3-30	6	CoV-2	CoV-2	Rogers et al. (39)
*ALC_2318830*	**AKVMTTYYYYYYGMDV**	**3-30**	**6**			
COVA2-14	ARVRYYDSSGYYEDY	1-69	4	CoV-1, CoV-2		Brouwer et al. (12)
*ALC_1780442*	**ARYDYYDSSGYYLDY**	**1-69**	**4**			
COV2-2270	AITYYYDSSGYWWDD	1-69	4	CoV-1, CoV-2		Zost et al. (38)
*ALC_1781971*	**ASTYYYDSSGYWFDY**	**1-69**	**4**			
COVA2-40	AGRYCSGGRCGWFDP	4-4	5	CoV-2		Brouwer et al. (12)
*ALC_1784026*	**ESRYCSGGSCGWFDP**	**4-4**	**5**			
COV2-2147	ARSTSGSYYYGMDV	3-30-3	6	CoV-1, CoV-2		Zost et al. (38)
CV34	ARSYGGSYYYGMDV	3-30-3	6	CoV-2		Seydoux et al. (40)
*ALC_1249094*	**ARGTRGSYYYGMDV**	**3-30-3**	**6**			
COV2-2006	ARPQSGGYYAPLDY	3-30-3	4	CoV-1, CoV-2		Zost et al. (38)
*ALC_1246650*	**ARPYSGSYYAPLDY**	**3-30-3**	**4**			
S304	ARGDSSGYYYYFDY	3-13	4	CoV-1, CoV-2	CoV-1, CoV-2	Pinto et al. (41)
*ALC_1245048*	**ARGYSSGYYYYFDY**	**3-13**	**4**			
COV2-2027	AIYGYYYYGLDV	3-30	6	CoV-2		Zost et al. (38)
*ALC_480504*	**AVYGYYYYGMDV**	**3-30**	**6**			

See **Supplementary Table 4** for convergent clonotypes with high CDRH3 homology to antibodies in the CoV-AbDab, but not sharing the same IGHV and IGHJ gene usage and allowing CDRH3 length mismatch. Each row represents a different group of similar sequences. Name: ID of the sequence. Black text represents the convergent clonotypes from this study, and blue text represents antibodies from CoV-AbDab. CDRH3, CDRH3 amino acid sequence. Green residues represent sequence matches between the convergent clonotype and the CoV-AbDab sequence, and red residues represent mismatches. IGHV: IGHV gene segment of the sequence. Green text represents matches between the convergent clonotype and the CoV-AbDab query sequence, and red text represents mismatches. IGHJ, IGHJ gene segment of the sequence. Green text represents matches between the convergent clonotype and the CoV-AbDab query sequence, and red text represents mismatches. ND, not determined. Binds/Neutralizes: Experimental confirmation of whether the sequence binds SARS-CoV-1 or SARS-CoV-2.

**Figure 4:**
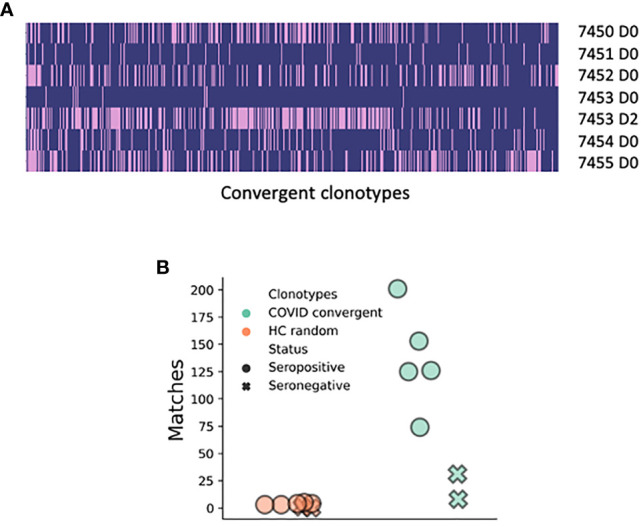
Matches of the 1,254 convergent clonotypes identified in the present study to the BCR sequence data from the six COVID-19 patients from Stanford, USA (15). **(A)** Plotted along the x-axis are the 463 convergent clonotypes represented in at least one sample from the Stanford study. Each row represents a separate BCR repertoire from the Stanford study; pink shading indicates that the convergent clonotype has a match. **(B)** Number of matches that the 1,254 convergent clonotypes have to the Stanford samples in comparison to the random selection of 1,254 clonotypes from the healthy controls.

Finally, we compared our data to a publicly available BCR deep sequencing dataset from six COVID-19 patients from Stanford, USA. Four hundred sixty-three of our 1,254 convergent clonotypes matched (using the same definition we used for clonotyping within our dataset) sequences in this dataset showing the high level of convergence between studies (**Figure 4A** and **Supplementary Table 5**). The average number of clonotype matches to the Stanford COVID-19 patient repertoires was 103, but this varied considerably between patients and timepoints. Two of the six patients were seronegative at the day of sampling (7451 and 7453), and these two patients had the fewest clonotype matches (8 and 31, respectively). Patient 7453 had an additional sample taken two days later (following seroconversion), and at this point had a large increase in the number of clonotype matches to 201. There was one of the 1,254 convergent clonotypes that was found across all six of the Stanford patients, and 2 clonotypes that were found in all five samples from seroconverted patients, but not found in the seronegative patients. In contrast, only 19 of the 1,254 healthy control clonotypes matched to sequences in the Stanford dataset (**Figure 4B**).

## Discussion

We have used deep sequencing of the BCR heavy chain repertoire to evaluate the B cell responses of 31 individuals with COVID-19. In agreement with previous studies, there was a skewing of the repertoire in the response to SARS-CoV-2 infection, with an increased use of certain V genes, an increase in the proportion of antibodies with longer CDRH3s, and an altered isotype subclass distribution (15). The significantly increased usage of IGHA1 observed in the COVID-19 patients is in line with mucosal responses, where the longer hinge in IGHA1 compared to IGHA2 may offer advantages in antigen recognition by allowing higher avidity bivalent interactions with distantly spaced antigens (42).

As anticipated, given the novel nature of the virus, SARS-CoV-2 infection largely stimulated a characteristically naïve-like response, rather than a reactivation of pre-existing memory B cells. There was an increased prevalence of unmutated, but class-switched BCR sequences, an increase in the diversity of BCRs present in the class-switched B cell compartment, and an increase in the usage of isotype subclasses that are associated with recent viral immunity. It is interesting that the levels of mutation were so low given that the time point at which we sampled these patients is on average 16 days since symptom onset, and therefore even longer since initial infection. In controlled vaccination studies, it has been seen that 10 days post novel antigen challenge are sufficient to generate high levels of mutation (43). Such expansion of unmutated but class-switched BCR sequences has however also been seen in humans following rotavirus (44) and dengue virus (45) infection. In mice infected with vesicular stomatitis virus, it is seen that there is an unmutated IgG response upon initial infection which confers protection, but secondary challenge results in increased mutation and a change in IGHV gene segment usage (46). These observations are attributed to natural, or autoreactive antibodies, which are produced rapidly upon initial viral infection and recognize repetitive structures such as viral capsids. The increased prevalence of autoreactive IGHV4-34 sequences in improving COVID-19 patients compared to stable or deteriorating COVID-19 patients further suggests a role for such natural or autoreactive antibodies in resolving infection and lowering the risk of pathology.

In addition to the naïve response, analysis of the largest clonal expansions in the SARS-CoV-2 patients, revealed them to be highly mutated, indicating that they may have arisen from memory recall. Such a secondary response to SARS-CoV-2 has been previously observed (47) and may be due to recall of B cells activated in response to previously circulating human coronaviruses, as recently highlighted (48, 49).

We observed a potential relationship between the observed repertoire characteristics and disease state. Improving patients showed a tendency toward a higher proportion of unmutated sequences compared to deteriorating patients, indicating that a naïve B cell response may be more effective than a memory recall response at clearing infection. Consistent with this hypothesis, Wec et al. found that B cells derived from memory recall generated low affinity, but SARS-CoV-1/2 cross-reactive antibodies, whereas B cells with the lowest mutation counts generated higher affinity SARS-CoV-2 specific antibodies (47). There is a clear need to expand on these findings by using larger sample cohorts and gathering more clinical data to aid understanding of the differences between individuals that respond with mild versus severe disease and have different recovery patterns. Building upon these observations could help to inform the future development of diagnostic assays to monitor and predict the progression of disease in infected patients.

A large number (1,254) of highly convergent clonotypes unique to COVID-19 were identified. Our approach of subtracting the convergent clonotypes also observed in healthy controls, and controls receiving influenza vaccination (22–24), allowed us to identify convergence specific to the disease cohort. The unbiased nature of the BCR repertoire analysis approach means that, while these convergent clonotypes are likely to include many antibodies to the spike protein and other parts of the virus they may also include other protective antibodies, including those to host proteins. Characterization of the heavy chains we have identified, coupled with matched light chains to generate functional antibodies will permit analysis of the binding sites and neutralizing potential of these antibodies. The report that plasma derived from recently recovered donors with high neutralizing antibody titers can improve the outcome of patients with severe disease (50), supports the hypotheses that intervention with a therapeutic antibody has the potential to be an effective treatment. A manufactured monoclonal antibody or combination of antibodies would also provide a simpler, scalable and safer approach than plasma therapy.

Sequence convergence between our 1,254 convergent clonotypes with heavy chains from published and patented SARS-CoV-1 and SARS-CoV-2 antibodies (30) supports several observations. Firstly, it demonstrates that our approach of finding a convergent sequence signature is a useful method for enriching disease-specific antibodies, as we find matches to known SARS-CoV spike-binding antibodies. Secondly, it shows that the clonotypes observed in response to SARS-CoV-2 overlap with those to SARS-CoV-1, presumably explained by the relatively high homology of the two related viruses (3). Indeed, here we show that there is an overrepresentation of clonotypes that correlate with patient clinical symptoms than is expected by chance, and these BCR sequences are associated with the dominant IgA1 and IgG1 responses. Finally, it shows that the convergence extends beyond our UK COVID-19 disease cohort.

Further evidence for convergence extending beyond our disease cohort came from the comparisons of our 1,254 convergent clonotypes to deep sequencing datasets from China (28) and the USA (15). The dataset from the USA is also from BCR sequencing of the peripheral blood of COVID-19 patients, and here we found matches to 463 of our 1,254 clonotypes. The dataset from China was from total RNA sequencing of the bronchoalveolar lavage fluid of SARS-CoV-2 infected patients. Only 16 unique CDRH3 sequences could be identified in this whole dataset, but one of them matched a convergent clonotype in the current study, showing that convergence can be seen both between different locations, and different sample types. We believe that the identification of such high BCR sequence convergence between geographically distinct and independent datasets could be highly significant and validates the disease association of the clonotypes, as well as the overall approach.

In summary, our BCR repertoire analysis provides information on the specific nature of the B cell response to SARS-CoV-2 infection. The information generated has the potential to facilitate the treatment of COVID-19 by supporting diagnostic approaches to predict the progression of disease, informing vaccine development and enabling the development of therapeutic antibody treatments and prophylactics.

## Data Availability Statement

Raw COVID-19 BCR sequence data are available under BioProject Accession PRJNA638224. Processed and annotated sequence data are available on the iReceptor AIRR Data Commons (52), and Zenodo (DOI: 10.5281/zenodo.3886395).

## Ethics Statement

The studies involving human participants were reviewed and approved by NHS HRA RES Ethics 19/SC/0361. The patients/participants provided their written informed consent to participate in this study.

## Author Contributions

JO, OC, AL, GT, DP, and PP conceived and designed the study. JG, SS, MR, RB-R, and AK conducted the data analysis with input from JO, RM, JD, GK, DF, LJ, RB-R, CD, OC, W-YL, GT, PP, and DP. GT, PP, and DP recruited COVID-19 participants and executed the clinical protocols. SS, JD, and W-YL processed the COVID-19 clinical samples. CC and JS recruited the breast cancer participants. SS and JD processed the breast cancer samples. JB oversaw sequencing of the libraries. JG, JO, GK, SS, and RM wrote the manuscript with input from all co-authors. All authors contributed to the article and approved the submitted version.

## Funding

MR is supported by an Engineering and Physical Sciences Research Council (EPSRC) and Medical Research Council (MRC) grant (EP/L016044/1). AK is supported by a Biotechnology and Biological Sciences Research Council (BBSRC) grant (BB/M011224/1).

## Conflict of Interest

JO, AL, OC, SS, JG, JD, RM, and DF are employees of Alchemab Therapeutics Limited. RB-R is a founder of and consultant to Alchemab Therapeutics Limited. GK is a consultant to Alchemab Therapeutics Limited. CC is a member of the AstraZeneca External Science Panel and has research grants from Roche, Genentech, AstraZeneca, and Servier that are administered by the University of Cambridge. JB was employed by Illumina, Inc.

The remaining authors declare that the research was conducted in the absence of any commercial or financial relationships that could be construed as a potential conflict of interest.
