# Supplementary material for: Deep Sequencing of B Cell Receptor Repertoires From COVID-19 Patients Reveals Strong Convergent Immune Signatures
Source: Front Immunol. 2020 Dec 15;11:605170. doi: 10.3389/fimmu.2020.605170 (PMC7769841; doi:10.3389/fimmu.2020.605170)
Supplement: Supplementary file 1 [file DataSheet_1.pdf]

Supplementary Material

Deep sequencing of B cell receptor repertoires from COVID-19 patients reveals strong convergent immune signatures

Jacob D. Galson[1*], Sebastian Schaetzle[1], Rachael J. M. Bashford-Rogers[1,2], Matthew I. J. Raybould[3], Aleksandr Kovaltsuk[3], Gavin J. Kilpatrick[1], Ralph Minter[1], Donna K. Finch[1], Jorge Dias[1], Louisa James[4], Gavin Thomas[4], Wing-Yiu Jason Lee[4], Jason Betley[5], Olivia Cavlan[1], Alex Leech[1], Charlotte M. Deane[3], Joan Seoane[6], Carlos Caldas[7], Daniel J. Pennington[4], Paul Pfeffer[4], Jane Osbourn[1]

[1] Alchemab Therapeutics Ltd, London, United Kingdom
[2] Wellcome Centre for Human Genetics, Oxford, United Kingdom
[3] Oxford Protein Informatics Group, Department of Statistics, University of Oxford, Oxford, United Kingdom
[4] Barts and The London School of Medicine and Dentistry, Queen Mary University of London, London, United Kingdom
[5] Illumina, Inc., Illumina Centre, Cambridge, United Kingdom
[6] Translational Research Program, Vall d'Hebron Institute of Oncology, Barcelona, Spain
[7] Cancer Research UK Cambridge Institute and Department of Oncology, Li Ka Shing Centre, University of Cambridge, Cambridge, United Kingdom

*** Correspondence:**
Jacob D. Galson
jake@alchemab.com

Keywords: COVID-19, SARS-CoV-2, B-cell repertoire, BCR, antibody, convergence

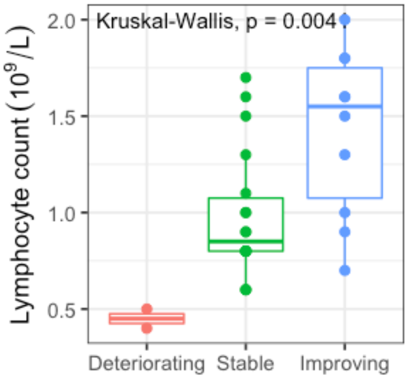

Supplementary Figure 1. Relationship between lymphocyte count and clinical status.

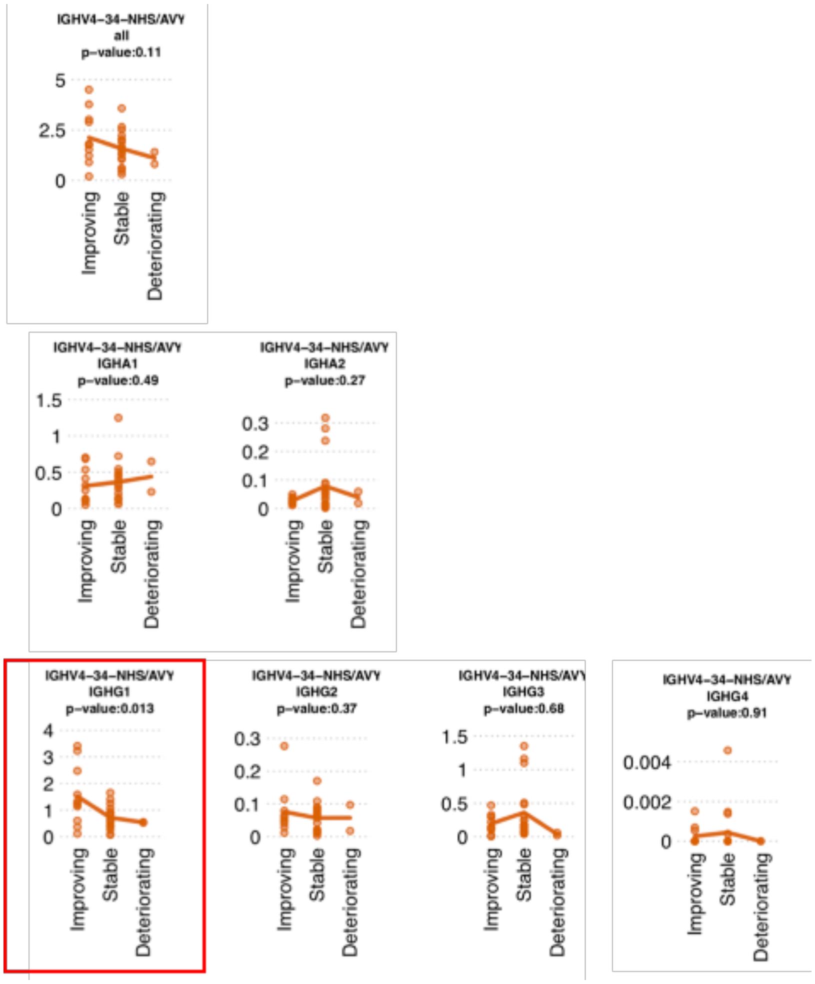

Supplementary Figure 2. The percentage of sequences containing the autoreactive "NHS and "AVY" motifs between COVID patients with improving, stable or worsening symptoms. IGHG1 (red box) was the only significant correlation. P-values are determined by MANOVA.

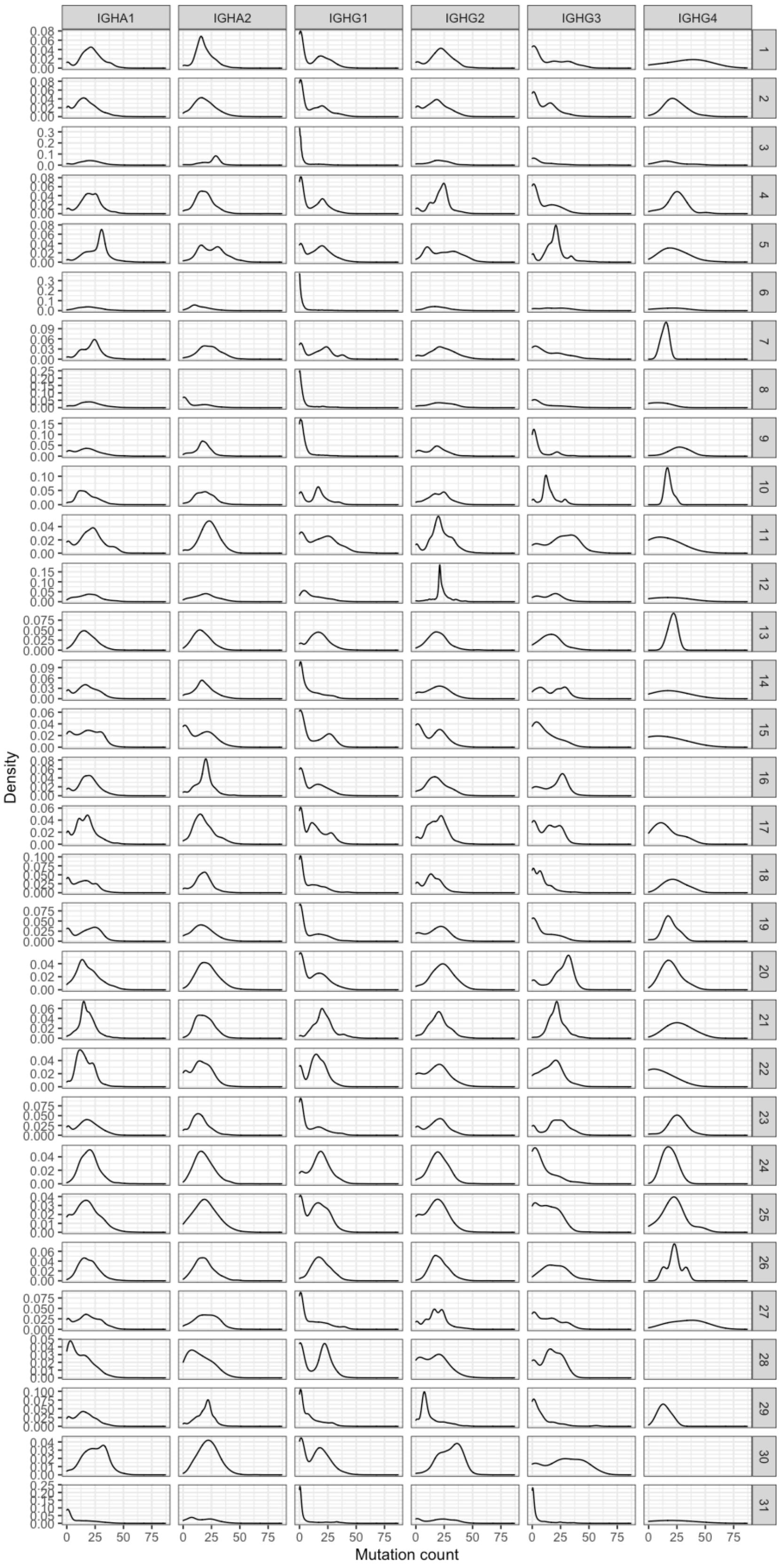

Supplementary Figure 3. Distribution of sequences with different numbers of mutations from germline. Each row is a different COVID-19 patient.

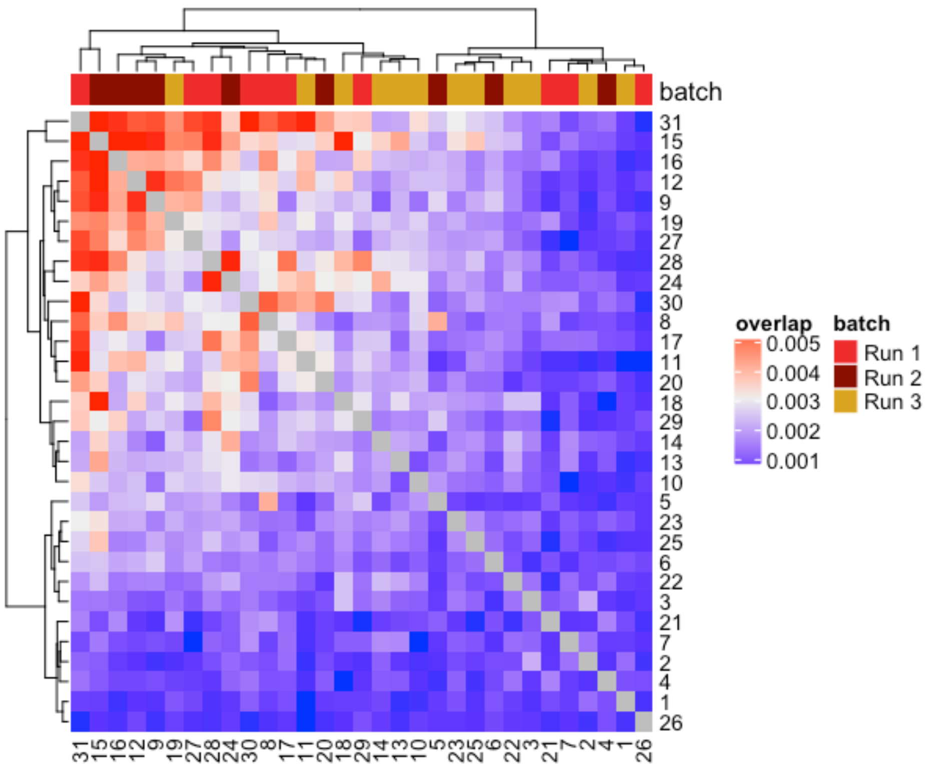

Supplementary Figure 4. Analysis of batch effects between the three sequencing runs. To normalise for different sequence numbers for each sample, a random subsample of 10,000 sequences per sample was taken. The clonotype convergence between each sample was then calculated using the jaccard index. Samples are hierarchically clustered in the heatmap based on this jaccard index.

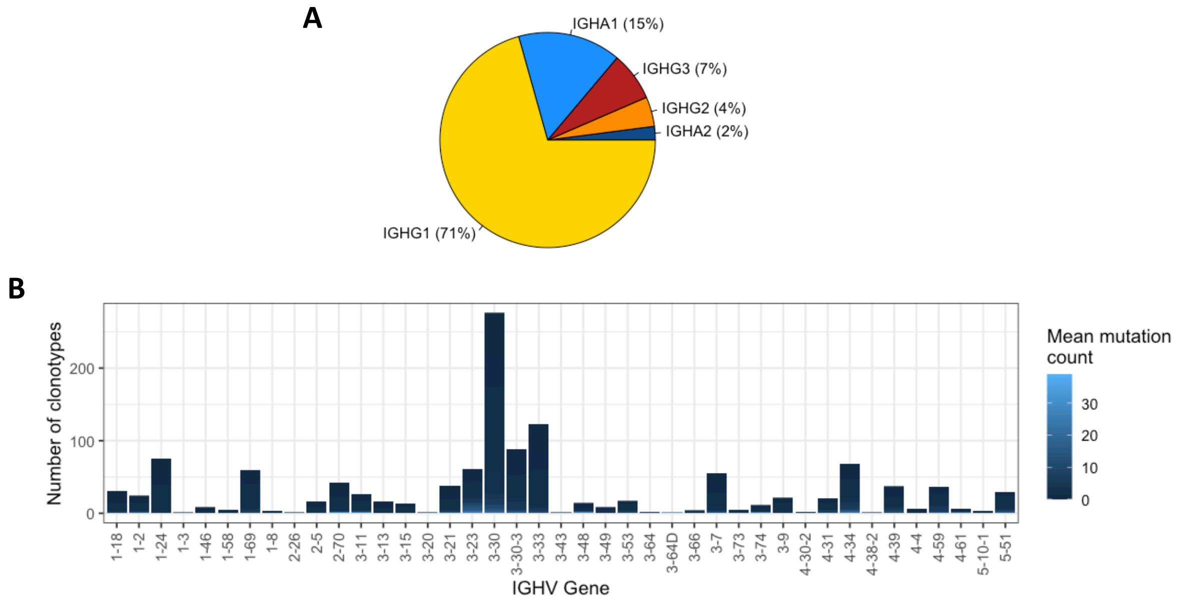

Supplementary Figure 5. Properties of the 1,254 convergent clonotypes **(A)** Isotype subclass usage of the sequences with the 1,254 convergent clonotypes. **(B)** IGHV gene segment usage of the 1,254 convergent clonotypes.

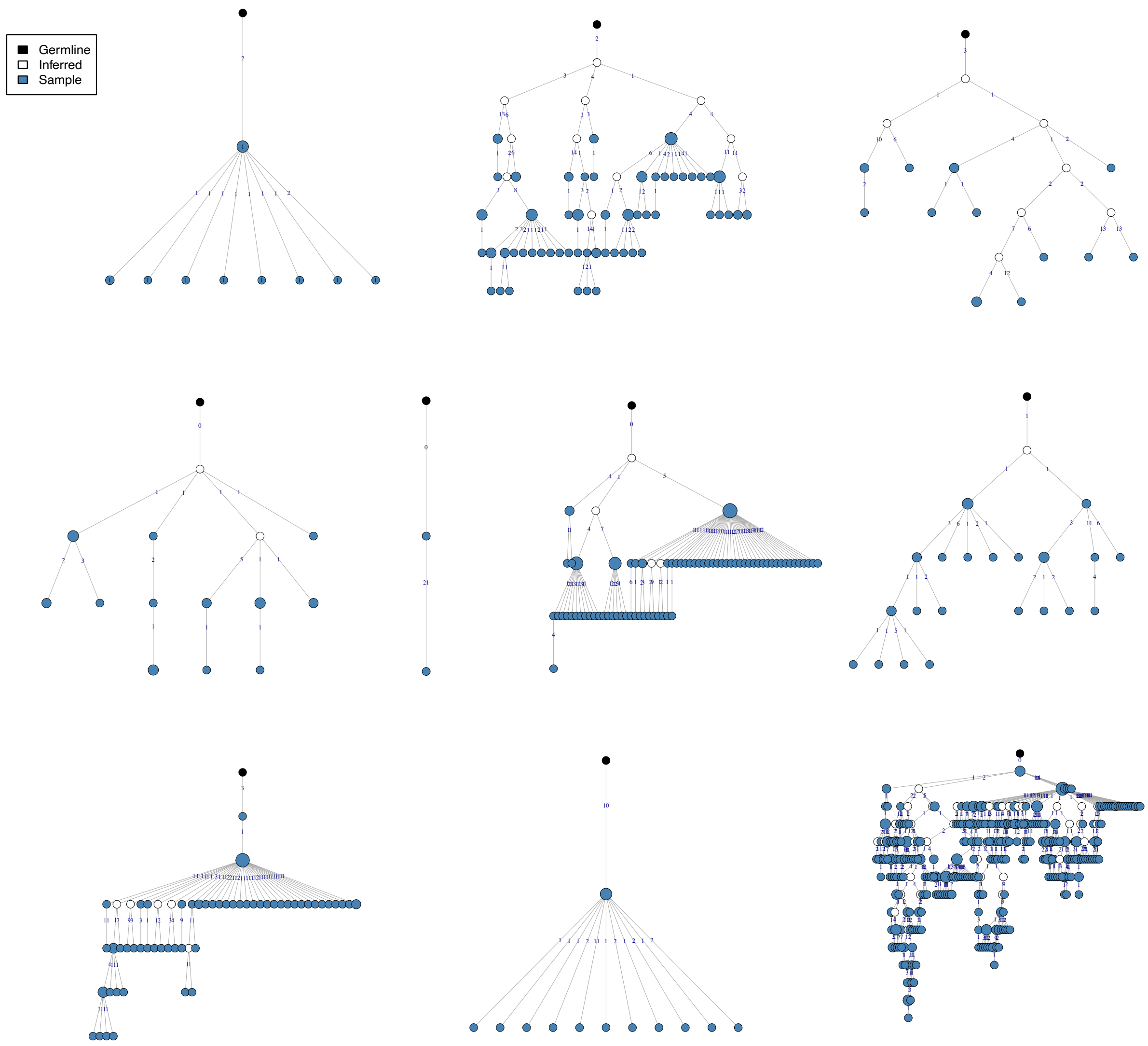

Supplementary Figure 6. Lineage trees of the convergent clonotype that matched to the bronchoalveolar lavage fluid data. Each lineage tree represents the members of the clonotype from each of the ten patients it was present in. Each node represents a unique sequence within the clonotype lineage tree, with the size indicative of the number of duplicate sequences present. Numbers on the edges of adjoining nodes show the number of mutations between the sequences.

Supplementary Table 1. Summary of number of unique sequences, and number of clonotypes obtained for each COVID-19 patient.

Participant ID	Gender	Age	Status	Unique BCR Sequences	Clonotypes
**1**	Female	48	Improving	184,613	30,438
**2**	Male	44	Stable	287,865	60,806
**3**	Male	66	Improving	169,645	21,928
**4**	Male	71	Improving	210,148	24,243
**5**	Male	86	Stable	235,013	34,368
**6**	Female	63	Stable	122,610	13,366
**7**	Female	82	Improving	217,356	21,004
**8**	Female	69	Stable	123,741	16,296
**9**	Female	57	Improving	194,778	20,779
**10**	Male	69	Stable	202,162	17,103
**11**	Male	69	Improving	152,272	17,144
**12**	Male	65	Improving	121,320	12,089
**13**	Female	33	Deteriorating	47,901	15,556
**14**	Male	40	Stable	257,725	53,649
**15**	Male	55	Stable	33,124	9,668
**16**	Male	78	Stable	37,168	10,828
**17**	Female	70	Stable	198,822	20,332
**18**	Male	39	Improving	233,408	26,604
**19**	Male	36	Improving	51,346	22,354
**20**	Male	52	Stable	39,325	9,475
**21**	Male	76	Deteriorating	221,975	18,588
**22**	Female	71	Stable	54,684	9,327
**23**	Male	26	Stable	202,995	41,493
**24**	Female	87	Stable	31,049	6,842
**25**	Male	29	Stable	41,025	14,840
**26**	Male	49	Stable	171,278	21,689
**27**	Male	35	Stable	280,496	36,937
**28**	Female	59	Stable	29,641	8,797
**29**	Female	42	Stable	253,163	35,211
**30**	Male	37	Improving	60,364	15,180
**31**	Male	40	Improving	329,223	78,400
			**Total:**	4,796,235	745,334

Supplementary Table 2. Summary of number of unique sequences, and number of clonotypes obtained for each healthy control subject. Only IGHA and IGHG data are included.

Participant ID	Gender	Age	Unique BCR Sequences	Clonotypes
**Co_C.001.2_PB**	Male	39	7,820	4,426
**Co_C.002.1_PB**	Male	35	43,813	12,481
**Co_C.003.1_PB**	Male	26	5,981	4,148
**Co_C.004.1_PB**	Female	27	10,098	5,203
**Co_C.005.1_PB**	Female	26	27,345	9,238
**Co_C.012.1_PB**	Female	26	17,204	12,198
**Co_C.013.1_PB**	Female	24	9,201	5,080
**Co_C.016.3_PB**	Female	25	8,792	3,991
**Co_C.017.2_PB**	Male	41	13,720	7,293
**Co_C.018.1_PB**	Male	33	6,079	4,268
**Co_C.020.1_PB**	Female	28	8,127	2,540
**Co_C.025.1_PB**	Male	30	56,489	18,265
**Co_C.026.1_PB**	Female	29	40,625	18,091
**Co_C.033.2_PB**	Female	15	21,652	5,194
**Co_C.038.1_PB**	Female	26	46,079	17,419
**Co_C.040.1_PB**	Male	20	140,640	27,263
**Co_C.042.1_PB**	Female	31	85,509	18,822
**Co_C.045.1_PB**	Female	51	35,540	8,038
**Co_C.046.1_PB**	Female	11	70,534	24,515
**Co_C.047.1_PB**	Female	18	148,395	20,784
**Co_C.048.1_PB**	Female	45	65,230	13,277
**Co_C.049.1_PB**	Female	25	97,538	23,486
**Co_C.051.1_PB**	Male	22	1,376	1,074
**Co_C.052.1_PB**	Male	15	9,466	4,126
**Co_C.055.1_PB**	Male	43	8,061	3,894
**Co_C.056.1_PB**	Female	30	9,048	4,659
**Co_C.060.1_PB**	Male	31	34,139	10,322
**Co_C.061.1_PB**	Male	36	12,578	6,016
**Co_C.062.1_PB**	Female	14	1,709	1,134
**Co_C.063.1_PB**	Male	20	15,348	6,424
**Co_C.065.1_PB**	Female	23	37,262	9,525
**Co_C.076.1_PB**	Female	34	7,068	2,792
**Co_C.077.1_PB**	Female	26	18,637	3,726
**Co_C.078.1_PB**	Female	26	15,383	3,505
**Co_P.166.1_PB**	Female	36	40,549	7,489
**Co_P.170.1_PB**	Female	45	41,564	14,183
**Co_P.178.1_PB**	Female	34	13,803	3,767
**Co_P.190.1_PB**	Female	11	4,565	2,245
**Co_P.191.1_PB**	Male	18	5,789	2,705
**Co_P.197.1_PB**	Female	15	99,308	19,654
		**Total:**	1,342,064	373,260

Supplementary Table 3. CDRH3 AA sequences identified from bronchoalveolar RNAseq data. Highlighted in green is the one identified in our SARS-CoV-2 patient dataset.

bestVHit	bestJHit	aaSeqCDR3
**IGHV2-26**	IGHJ3	CARDSGRHLGPFDIW
**IGHV1-2**	IGHJ3	CATPYYYDGGLDAFDIW
**IGHV3-74**	IGHJ5	CARDLSRTNWFDPW
**IGHV3-15**	IGHJ4	CTTDLHDYGDSDYW
**IGHV3-15**	IGHJ4	CTTDFGGMITFGGVLRRI
**IGHV3-21**	IGHJ4	CARAQSRGGYDSFFDFW
**IGHV3-21**	IGHJ4	CGRGGPGTGIDYW
**IGHV4-59**	IGHJ5	CARGGQYNNWFAPW
**IGHV3-74**	IGHJ5	CVRDLSRTNWFDPW
**IGHV3-15**	IGHJ4	YTRDLHDYGDSDYW
**IGHV3-23**	IGHJ3	CAKIPSFLSDYDVHPNDAIDIW
**IGHV5-10-1**	IGHJ4	CARHPQGAQFSNLGTYYFDYW
**IGHV4-59**	IGHJ4	CARDGEYGGLAMW
**IGHV5-51**	IGHJ6	CARPGTYYDILTGYSNHGMDVW
**IGHV4-39**	IGHJ5	CARHASFRGTNYNWFDPW
**IGHV3-53**	IGHJ5	CARDTSTEDVAWWFDPW

Supplementary Table 4. Convergent clonotypes with high CDRH3 homology to antibodies in the CoV-AbDab. Name: ID of the sequence. Black text represents the convergent clonotypes from this study, and blue text represent antibodies from CoV-AbDab. Highlighted in yellow are those which would be considered to belong to the same clonotype and are represented in Table 1. CDRH3: CDRH3 amino acid sequence. Green residues represent sequence matches between the convergent clonotype and the CoV-AbDab sequence, and red residues represent mismatches. IGHV: IGHV gene segment of the sequence. Green text represents matches between the convergent clonotype and the CoV-AbDab query sequence, and red text represents mismatches. IGHJ: IGHJ gene segment of the sequence. Green text represents matches between the convergent clonotype and the CoV-AbDab query sequence, and red text represents mismatches. ND – not determined. Binds/Neutralises: Experimental confirmation of whether the sequence binds SARS-CoV-1 or SARS-CoV-2.

Name	CDRH3	IGHV	IGHJ	Binds	Neutralises	Reference
C154	AKQ-AGPYCSGGSCYSAPFDY	3-30	4	CoV-1, CoV-2	CoV-2	Robbiani et al., 2020
ALC_3983948	**AKV-SGPYCSGGSCYSFYFDY**	**3-30**	**4**			
ALC_4259294	**AKMGGGPYCSGGSCYSNYFDY**	**3-30**	**4**			
COV2-2901	ARSYDILTGYRDAFDI	3-53	3	CoV-2		Zost et al., 2020
COV2-2068	ARSYDILTGYRDAFDI	3-53	3	CoV-2	CoV-2	
COV2-2767	ARSYDILTGYRDAFDI	3-53	3	CoV-2		
COV2-2790	ARSYDILTGYRDAFDI	3-53	3	CoV-2	CoV-2	
ALC_2318471	**VRNYDILTGYSDAFDI**	**3-53**	**3**			
COV2-2007	AKVSATYYYYYYGMDV	3-30	6	CoV-1, CoV-2		Zost et al., 2020
CC12.17	AKSSGSYYYYYYGMDV	3-30	6	CoV-2	CoV-2	Rogers et al., 2020
ALC_2318830	**AKVMTTYYYYYYGMDV**	**3-30**	**6**			
REGN10977	ARTPFYYDSSGYYLDY	1-69	4	CoV-2	CoV-2	Hansen et al., 2020
COVA2-14	ARVR-YYDSSGYYEDY	1-69	4	CoV-1, CoV-2		Brouwer et al., 2020
ALC_1780442	**ARYD-YYDSSGYYLDY**	**1-69**	**4**			
COV2-2270	AITYYYDSSGYWWDD	1-69	4	CoV-1, CoV-2		Zost et al., 2020
ALC_1781971	**ASTYYYDSSGYWFDY**	**1-69**	**4**			
COVA2-40	AGRYCSGGRCGWFDP	4-4	5	CoV-2		Brouwer et al., 2020
ALC_1784026	**ESRYCSGGSCGWFDP**	**4-4**	**5**			
COV2-2147	ARSTSGSYYYGMDV	3-30-3	6	CoV-1, CoV-2		Zost et al., 2020
COV2-2341	ARSTSGSYYYGMDV	3-30-3	6	CoV-1, CoV-2		
COV2-2160	ARSTSGSYYYGMDV	3-30-3	6	CoV-1, CoV-2		
COV2-2159	ARSTSGSYYYGMDV	3-30-3	6	CoV-1, CoV-2		
CV27	ARSFGGSYYYGMDV	3-30	6	CoV-2		Seydoux et al., 2020
CV34	ARSYGGSYYYGMDV	3-30-3	6	CoV-2		
ALC_1248726	**ARAYSGSYYYGMDV**	**3-53**	**6**			
ALC_1249094	**ARGTRGSYYYGMDV**	**3-30-3**	**6**			
ALC_1255857	**ARGFGGSYYYGMDV**	**3-53**	**6**			
ALC_1251900	**ARAYSGSYYYGMDV**	**3-7**	**6**			
COV2-2639	ARAGGGSYRGPFDY	3-30	4	CoV-1, CoV-2		Zost et al., 2020
2M-14E5	ARSGGGSYRGPFDY	3-30	4	CoV-2		
ALC_1245591	**ARVIGGSYRGPFDY**	**3-30-3**	**4**			
COV2-2006	ARPQSGGYYAPLDY	3-30-3	4	CoV-1, CoV-2		Zost et al., 2020

ALC_1246650	ARPYSGSYYAPLDY	3-30-3	4			
S304	ARGDSSGYYYYFDY	3-13	4	CoV-1, CoV-2	CoV-1, CoV-2	Pinto et al., 2020
ALC_1245048	ARGYSSGYYYYFDY	3-13	4			
COV2-2027	AIYGYYYYGLDV	3-30	6	CoV-2		Zost et al., 2020
ALC_480504	AVYGYYYYGMDV	3-30	6			
ALC_481016	ASYGYYYYGMDV	3-30-3	6			
COVA2-04	ARDLERAGGMDV	3-53	6	CoV-2	CoV-2	Brouwer et al., 2020
ALC_498298	ARDLEAAGGMDV	3-66	6			
CA1	AREGYCSGGSCYSGYYYYYGMDV	1-18	6	CoV-2	CoV-2	Shi et al., 2020
ALC_4634307	AREGYCSGGSCYTDYYYYYGMDV	3-7	6			
ALC_4258298	ARDTYCSGGSCY--YYYYYGMDV	3-7	6			
COV2-2445	ARRRSSSRYSSGWYMYYYYMDV	3-33	6	CoV-2		Zost et al., 2020
COV2-2173	ARRRSSSRYSSGWYMYYYYMDV	3-20	6	CoV-2		
COV2-2191	ARRRSSSRYSSGWYMYYYYMDV	3-20	6	CoV-2		
ALC_4259233	ARDVSSSWYSSGWY-YYYYMDV	3-7	6			
COVA1-19	ARWKS-DYYDSSGYYPAAFDI	3-21	3	CoV-2		Brouwer et al., 2020
ALC_4259226	ARWRRTYYYDSSGYYPDAFDI	3-21	3			
C150	ARPTAVAAAGNYFYYYGMDV	3-74	6	CoV-2		Robbiani et al., 2020
ALC_3663937	ARDSGVAAAGNY-YYYGMDV	3-7	6			
12E	ARVGYYGDYAWGYYYYGMDV	1-18	6	CoV-1	CoV-1	WO2005060520
ALC_3293962	ARH--YGDYVFGYYYYGMDV	4-39	6			
256	ARDGYGSGS-DYYYYYYMDV	3-30	6	CoV-1	CoV-1	Sui et al., 2006
ALC_3985973	ARGGYGSGSYYNYYYYYMDV	1-69	6			
CV23	ARV-WGYCSGGSCYVDAFDI	1-3	3	CoV-2		Seydoux et al., 2020
ALC_3664160	ARVGLGYCSGGSCY-SAFDI	4-34	3			
COV2-2146	ARRSYRSSWYYYYGMDV	3-48	6	CoV-2		Zost et al., 2020
COV2-2263	ARRSYRSSWYYYYGMDV	3-48	6	CoV-2		
ALC_2822775	ARVGYSSSWYYYYGMDV	1-69	6			
0317-A9	ATATAMDGYYYYYGMDV	1-24	6	CoV-2		Chi et al., 2020
ALC_2317864	AVNTAMD-YYYYYGMDV	3-21	6			
COV2-2844	ARAQGGN-YYYGMDV	3-30-3	6	CoV-1, CoV-2		Zost et al., 2020
COV2-2564	ARAQGGN-YYYGMDV	3-30-3	6	CoV-1, CoV-2		
ALC_1782084	ARGQGGSQYYYGMDV	4-54	6			
COVA3-01	ARGPAATYYYYMDV	4-59	6	CoV-1, CoV-2		Brouwer et al., 2020
COVA3-09	ARGPAATYYYYMDV	3-9	6	CoV-2		
ALC_1244470	ATGVAATYYYYMDV	4-61	6			
COV2-2832	ARGDGGYYSPFDY	3-66	4	CoV-2	CoV-2	Zost et al., 2020
ALC_815774	ARSRGGYYSPFDY	3-30-3	4			
COV2-2919	ARTMATINAFDI	2-70	3	CoV-2	CoV-2	Zost et al., 2020
ALC_482575	ARIMATINAFDI	2-70	3			
COV2-2178	ARVGSSSWYFDY	3-7	4	CoV-1, CoV-2		Zost et al., 2020
ALC_482648	ARVPSSSWYFDY	3-11	4			
C109	AIQLWLRGGYDY	3-7	4	CoV-2		Robbiani et al., 2020
ALC_481831	AVQLWLRGGFDY	4-59	4			

BD-494	ARDLVVYGMDV	3-53	6	CoV-2	CoV-2	Cao et al., 2020
BD-498	ARDLVVYGMDV	3-53	6	CoV-2	CoV-2	
BD-505	ARDRVVYGMDV	3-53	6	CoV-2	CoV-2	
BD-507	ARDLVVYGMDV	3-53	6	CoV-2	CoV-2	
ALC_265226	**ARVLTVYGMDV**	**3-33**	**6**			
BD-604	ARDLGPYGMDV	3-53	6	CoV-2	CoV-2	Du et al., 2020
ALC_269736	**ARGAGPYGMDV**	**3-11**	**6**			
C210	ARDLMAYGMDV	3-53	6	CoV-2	CoV-2	Robbiani et al., 2020
ALC_261066	**ARDSTAYGMDV**	**4-34**	**6**			
CV10	ARGFDY	4-59	4	CoV-1, CoV-2		Seydoux et al., 2020
ALC_7192	**ARGFDY**	**1-2**	**4**			
ALC_7213	**ARGFDY**	**1-18**	**4**			
ALC_7353	**ARGFDY**	**5-51**	**4**			
ALC_7448	**ARGFDP**	**4-34**	**5**			

Supplementary Table 5. Details of the 463 convergent clonotypes that matched to samples in the Nielsen *et al*. data.

Cluster Centre CDRH3	IGHV gene	IGHJ gene	Total Matches	Seropositive Matches	Seronegative Matches
CARRFDYW	3-23	4	6	5	1
CARGFDYW	1-18	4	5	5	0
CARGFDPW	4-34	5	5	5	0
CARVFDYW	3-30	4	5	4	1
CARVNSGSYLGAFDIW	3-30	3	5	4	1
CARGFDYW	1-2	4	4	4	0
CAREVLVYFDYW	3-33	4	4	4	0
CARVLSYYGMDVW	3-23	6	4	4	0
CARSSGYYYFDYW	3-30	4	4	4	0
CARVVSYYYGMDVW	3-21	6	4	4	0
CARVASYYYGMDVW	3-23	6	4	4	0
CTRNDFWSGYYFDYW	3-7	4	4	4	0
CARTYSGSYTDAFDIW	3-30	3	4	4	0
CAREGVVGATTGLDYW	3-30	4	4	4	0
CARDGVVTATTGLDYW	3-33	4	4	4	0
CARDRIVGGTTGLDYW	3-33	4	4	4	0
CTTVVYYYDSSGYSNDAFDIW	3-15	3	4	4	0
CARGYSSGWYFDYW	3-30	4	4	3	1
CARVPSSSWYFDYW	3-11	4	4	3	1
CTTGGRLW	3-15	4	3	3	0
CASGFDYW	1-69	4	3	3	0
CARGVDPW	1-2	5	3	3	0
CARDVGAQFDYW	3-30	4	3	3	0
CARDLYTFGMDVW	3-53	6	3	3	0
CAVYGYYYYGMDVW	3-30	6	3	3	0
CAKVRGGYYYFDYW	3-30	4	3	3	0
CAHRRSYYDAFDIW	2-5	3	3	3	0
CTSNDFWSGYSDYW	3-49	4	3	3	0
CARLGGTAWYFDLW	3-7	2	3	3	0
CATDGIVGATGLDYW	3-33	4	3	3	0
CAVPRGYSYGPFDYW	3-48	4	3	3	0
CTRGDFWSGYWSDYW	3-49	4	3	3	0
CSRPYSGSYYSYFDYW	3-30	4	3	3	0
CARTYSGSYYSWFDPW	3-30-3	5	3	3	0
CARVRSGSYYDAFDIW	3-30-3	3	3	3	0
CARVRGGSYYGVFDYW	3-30	4	3	3	0
CAKTVSGSYYSPFDYW	3-30	4	3	3	0
CARVPGGSYYGPFDYW	3-30	4	3	3	0
CARSNSGSYYHAFDIW	3-30-3	3	3	3	0
CARVSSGSYFGAFDIW	3-30-3	3	3	3	0
CATARAGSYYYGMDVW	3-23	6	3	3	0
CAKVRGGSYRDAFDIW	3-30	3	3	3	0
CASSGSGSYYSPFDYW	3-30	4	3	3	0
CARTYSGSYYGAFDIW	3-23	3	3	3	0
CAKTYSGSYYSSFDYW	3-30	4	3	3	0
CARAYSGSYYYGMDVW	3-7	6	3	3	0
CARAYSGSYWAAFDIW	3-30	3	3	3	0
CARGYYDSSGYYNWFDPW	3-13	5	3	3	0

CARGRTVTTLYYYYGMDVW	4-39	6	3	3	0
CARDSVLRYFDWTYYYYGMDVW	3-11	6	3	3	0
CARVGYCSSTSCYSYYYYGMDVW	3-33	6	3	3	0
CARGRPLDAFDIW	4-31	3	3	2	1
CARDSTAYGMDVW	4-34	6	3	2	1
CASVHSSSWFFDYW	3-7	4	3	2	1
CARSGSYYGPFDYW	3-30	4	3	2	1
CAKDSYSSSWYFDYW	3-30	4	3	2	1
CARDSGPYYYGMDVW	3-30-3	6	3	2	1
CARGFNYYDSSGFDYW	3-30	4	3	2	1
CERDRSVCSSTSCYYYYGMDVW	3-30	6	3	2	1
CARGRYYYDSSGYSYYYYGMDVW	1-69	6	3	2	1
CATSGYW	1-24	4	2	2	0
CTTWGGYW	3-15	4	2	2	0
CAKGVDVW	3-23	6	2	2	0
CAQGLDYW	4-59	4	2	2	0
CAMGFDDW	4-59	4	2	2	0
CAKSDGMDVW	3-9	6	2	2	0
CAKDVGGNYGYW	3-23	4	2	2	0
CAREYLVDLDYW	3-33	4	2	2	0
CARTPGPYFDYW	3-30	4	2	2	0
CARVVYDYFDYW	3-53	4	2	2	0
CARGYRDYGDYW	3-33	4	2	2	0
CARVLSYYGMDVW	4-34	6	2	2	0
CARMTTVTTYDYW	2-70	4	2	2	0
CARYSSSSDFDYW	1-69	4	2	2	0
CARAVNFGWFDPW	1-18	5	2	2	0
CVRDWGEYYFDYW	3-53	4	2	2	0
CARGVWDLWYFDLW	4-34	2	2	2	0
CARQQWLRGRFDYW	4-39	4	2	2	0
CARETGSGSYPDYW	3-33	4	2	2	0
CARVLGTYYYMDVW	3-30	6	2	2	0
CARDLSYGDYNDYW	3-33	4	2	2	0
CATLVDYYYYMDVW	3-9	6	2	2	0
CARGGGYGHYFDYW	3-30-3	4	2	2	0
CVGELLPYYGMDVW	3-33	6	2	2	0
CAVQLWLRGGFDYW	4-59	4	2	2	0
CAKVMAVAGTGDYW	3-30	4	2	2	0
CARVLVPVYGMDVW	3-30	6	2	2	0
CARVLSPLYGMDVW	3-33	6	2	2	0
CARDYGDYGGLDYW	3-30	4	2	2	0
CSKDIYGSGSYPDYW	3-9	4	2	2	0
CVTPRGYSYGPFDYW	3-21	4	2	2	0
CATGGRYYYYGMDVW	3-30	6	2	2	0
CARDPVAVAGPFDYW	3-30	4	2	2	0
CAREGVGATYYFDYW	3-30	4	2	2	0
CARVQGGYYYGMDVW	3-30	6	2	2	0
CAKGYSSSWYFFDYW	3-9	4	2	2	0
CATPRVYSYGPFDYW	3-23	4	2	2	0
CARTSGSYYSPFDYW	3-30-3	4	2	2	0
CARDREAVAGPFDYW	3-30	4	2	2	0
CAREGVGATYYFDYW	3-33	4	2	2	0
CTRTDFWSGYYPDDW	3-49	4	2	2	0

CAKDVGYSSGLPDYW	3-9	4	2	2	0
CTRYDFWSGYFSDYW	3-49	4	2	2	0
CARGYSSYYWYFDLW	3-30	2	2	2	0
CAREGIVGATGLDYW	3-30	4	2	2	0
CATLRGGYYYGMDVW	3-9	6	2	2	0
CASPRSGSYYGAFDIW	3-30-3	3	2	2	0
CARTKSGSYWYYFDYW	3-30	4	2	2	0
CARVRSGSYLSHFDYW	3-30	4	2	2	0
CARVSSGSYYGWFDYW	3-30-3	4	2	2	0
CARDRSGSYYHGMDVW	3-30	6	2	2	0
CASSYSGSYLSAFDIW	3-30-3	3	2	2	0
CVRAVAGTYYYGMDVW	3-30	6	2	2	0
CSRSGSGSYYGWFDPW	3-30-3	5	2	2	0
CATSPATVTTGWFDPW	1-24	5	2	2	0
CARGSYGSYYYGMDVW	3-30	6	2	2	0
CARVYSGSYFSPFDYW	3-30-3	4	2	2	0
CARVYGGSYLGAFDIW	3-30	3	2	2	0
CAKTNSGSYYYGMDVW	3-30	6	2	2	0
CAKGPYGSGSYYVDYW	3-9	4	2	2	0
CARGPRGYSYGYVDYW	3-21	4	2	2	0
CAREGDYYYYYGMDVW	3-33	6	2	2	0
CAREGVITGTTGLDYW	3-33	4	2	2	0
CARVSGGSYYGNFDYW	3-30-3	4	2	2	0
CARVRGGSYWGDFDYW	3-30	4	2	2	0
CAREGQTTVTTGIDYW	3-33	4	2	2	0
CATTSIVGATSWFDPW	1-24	5	2	2	0
CARQEDYYDSSGFDYW	3-33	4	2	2	0
CARTRSGSYLYYFDYW	3-30	4	2	2	0
CARAYSGSYFNWFDPW	3-30-3	5	2	2	0
CARDYSGSYLGLFDYW	3-30-3	4	2	2	0
CARPYSGSYYWWFDPW	3-23	5	2	2	0
CARDVAVAGDDAFDIW	3-33	3	2	2	0
CARTRGGGYFDAFDIW	3-30-3	3	2	2	0
CAKGVSGYYLDAFDIW	3-30	3	2	2	0
CARTYSGSYRNWFDPW	3-33	5	2	2	0
CARVPGGSYFGAFDIW	3-30-3	3	2	2	0
CARVYGGSYIGYFDYW	3-30	4	2	2	0
CARETLDSTSSDFDYW	3-30	4	2	2	0
CARGSSGNYCYGMDVW	3-30	6	2	2	0
CARRNSSGWYGDWFDPW	2-5	5	2	2	0
CARATMIVVVVGAFDIW	4-31	3	2	2	0
CSKDRPYDYVWGSLDYW	3-30	4	2	2	0
CAKDWGYDYGDYGPDYW	3-30	4	2	2	0
CATRYCSGGSCSLFDYW	4-4	4	2	2	0
CARYDYYDSSGYYLDYW	1-69	4	2	2	0
CTKADCSSTSCQNWFDPW	3-9	5	2	2	0
CAKVGYSYGYPVYYFDYW	3-30	4	2	2	0
CARVSYDSSGYYYYFDYW	3-13	4	2	2	0
CARGGYSYGYYYYGMDVW	3-53	6	2	2	0
CARSPITMIVVVNAFDIW	4-34	3	2	2	0
CAREGTYCGGDCYSGLDYW	3-33	4	2	2	0
CARTYYDILTGHNYGMDVW	2-70	6	2	2	0
CARDSYSSSSYYYYYGMDVW	3-7	6	2	2	0

CARGRYSSSWYGVRNWFDPW	4-59	5	2	2	0
CAKVSGPYCSGGSCYSFYFDYW	3-30	4	2	2	0
CARGWPSSSWYEYYYYYGMDVW	4-34	6	2	2	0
CAKVVGVYCSGGSCYGGYFDYW	3-30	4	2	2	0
CTRGVPGYW	4-34	4	2	1	1
CARSDGYNFDYW	4-59	4	2	1	1
CARDGGVLFDYW	3-23	4	2	1	1
CARDTSGWYLDYW	3-30-3	4	2	1	1
CARVLSYYGMDVW	3-30-3	6	2	1	1
CAGGSYYYGMDVW	3-33	6	2	1	1
CARDRGYSYGSFDYW	3-33	4	2	1	1
CARVLSGWYVGWFDPW	1-69	5	2	1	1
CARVYSGSYYPAFDIW	3-21	3	2	1	1
CAKGSWYSSSWYYFDYW	3-30	4	2	1	1
CAKDRSGSYGYYGMDVW	3-30	6	2	1	1
CARGGYSSSWYYYYGMDVW	3-13	6	2	1	1
CARGFDYW	5-51	4	1	1	0
CARGFVLW	4-59	4	1	1	0
CAKGGDMDVW	3-30	6	1	1	0
CAKVGGYFDYW	3-9	4	1	1	0
CASWVNWFDPW	4-34	5	1	1	0
CTSLSAPTDYW	3-74	4	1	1	0
CPRGLEWELLNW	3-33	4	1	1	0
CARESGSGLDYW	3-30-3	4	1	1	0
CARWGGSGLDYW	3-30	4	1	1	0
CARSVVSYFDYW	3-53	4	1	1	0
CATSFYYYMDVW	3-21	6	1	1	0
CARGGGDYVDYW	3-30-3	4	1	1	0
CARDVGGYFDLW	3-30	2	1	1	0
CARYHGSALDYW	3-30-3	4	1	1	0
CAKEGIVLLDYW	3-30	4	1	1	0
CARDSGSYLGWW	3-30	4	1	1	0
CASLAGSGFDYW	3-30-3	4	1	1	0
CARYSSGYYFDYW	3-21	4	1	1	0
CATEGLLDAFDIW	3-30	3	1	1	0
CARVLSGYGFDYW	3-30	4	1	1	0
CARDPYGGGFDYW	3-30-3	4	1	1	0
CARDGLSYGLDYW	3-30	4	1	1	0
CARDSYSSGLDYW	3-7	4	1	1	0
CAREGSGWTIDYW	3-30	4	1	1	0
CAKVAAAGTTDYW	3-9	4	1	1	0
CAREHGDYGFDYW	3-7	4	1	1	0
CVGELPYYGMDVW	3-30	6	1	1	0
CARNGGNNWFDPW	3-30-3	5	1	1	0
CARDGTLEPLDYW	1-69	4	1	1	0
CGRGYGDYWFDPW	3-53	5	1	1	0
CARDLYDDAFDIW	3-66	3	1	1	0
CARRYGVRPFDYW	2-70	4	1	1	0
CARGAGPYGMDVW	3-11	6	1	1	0
CVRARDGYNHFDYW	3-30-3	4	1	1	0
CAKVSSYGYYFDYW	3-23	4	1	1	0
CATSLLWFGELLYW	3-30	4	1	1	0
CARGSSGYYYFDYW	3-30-3	4	1	1	0

CAKVYRSGNAFDIW	3-23	3	1	1	0
CTGSTGYYYGMDVW	3-33	6	1	1	0
CARSDGYNSYFDYW	3-30	4	1	1	0
CARWLGGYYGMDVW	3-30-3	6	1	1	0
CSRDYGDYGGMDVW	3-30	6	1	1	0
CVREDYGDFGFDYW	3-30	4	1	1	0
CARDYRFGELGDYW	3-33	4	1	1	0
CASSSSPYYGMDVW	3-33	6	1	1	0
CAVGLGYYYGMDVW	3-23	6	1	1	0
CARDWAVGATTDYW	3-33	4	1	1	0
CARRPVGVTPFDYW	5-51	4	1	1	0
CAKMYGSGSYIDYW	3-9	4	1	1	0
CARGGTLYDAFDIW	3-30-3	3	1	1	0
CARSYSGYVPFDYW	2-70	4	1	1	0
CARGWGGYYYFDYW	3-30	4	1	1	0
CARTKTGYYYFDYW	3-30	4	1	1	0
CARIMATINAFDIW	2-70	3	1	1	0
CASTTYYYYYMDVW	1-18	6	1	1	0
CARDVAVGDAFDSW	3-33	3	1	1	0
CVRVVAATVSFDYW	2-70	4	1	1	0
CAKLTYGDSPFDYW	3-9	4	1	1	0
CASGSGSYYYFDYW	3-30	4	1	1	0
CARYTGDYYGMDVW	5-51	6	1	1	0
CARDGGLAAAFDYW	3-33	4	1	1	0
CATELLWRYGMDVW	3-30	6	1	1	0
CAKDPYGDLAFDYW	3-23	4	1	1	0
CARDGGLVRGVDYW	3-33	4	1	1	0
CTRDDFRSGYTNYW	3-49	4	1	1	0
CARELSGWYGFDYW	3-33	4	1	1	0
CARDRRLAEEFDYW	3-33	4	1	1	0
CARDGTVTRTFDYW	3-33	4	1	1	0
CTQDDFWSGYGHYW	3-49	4	1	1	0
CGRDEYSSGWYFDYW	3-33	4	1	1	0
CAKGRGYSYGYFDYW	3-30	4	1	1	0
CTTGSGSYLGWFDPW	1-24	5	1	1	0
CASGYSGSYWYFDYW	3-30	4	1	1	0
CVLGSGSYYTPFDYW	3-30	4	1	1	0
CARIYSSSWPSFDYW	2-70	4	1	1	0
CARDSRGYSYGFDYW	3-33	4	1	1	0
CARGGIAVADAFDIW	3-30	3	1	1	0
CERAVVAAHNWFDPW	3-21	5	1	1	0
CARGPRSSSDYFDYW	3-23	4	1	1	0
CARDYLGYSYGSDYW	3-7	4	1	1	0
CARVLVVNYYGMDVW	3-30	6	1	1	0
CARDSIVSGWLFDYW	3-33	4	1	1	0
CARQLTYYYYGMDVW	3-30	6	1	1	0
CTRYDFWGGYYVDYW	3-49	4	1	1	0
CARTSSGYYSKFDYW	3-30	4	1	1	0
CARVGGIAVAGLDYW	3-30	4	1	1	0
CARDLSGSYTYFDYW	3-30	4	1	1	0
CATELLYYYYGMDVW	3-33	6	1	1	0
CARDQRSSGWYVDYW	3-33	4	1	1	0
CARTWAAAGLGADYW	2-70	4	1	1	0

CARSRGGYYSPFDYW	3-30-3	4	1	1	0
CARESVAALDAFDIW	3-30	3	1	1	0
CARARGGSYSYFDYW	3-30	4	1	1	0
CARGYGDYVPNFDYW	4-34	4	1	1	0
CAREGVTATVYFDYW	3-74	4	1	1	0
CARESIVVVPAADYW	3-74	4	1	1	0
CSRNYYDSSDAFDIW	3-30	3	1	1	0
CARTLVLSYYGMDVW	3-33	6	1	1	0
CARVLVLYYYGMDVW	3-30-3	6	1	1	0
CATNDYYYYYYMDVW	3-33	6	1	1	0
CVRDMSGSYYGPFDYW	3-30	4	1	1	0
CATVHSGSYLGYFDYW	3-30	4	1	1	0
CASAGSGSYRGWFDPW	3-30	5	1	1	0
CPRGYSGSYFSAFDIW	3-30	3	1	1	0
CARVYSGSYYSHFDYW	3-30	4	1	1	0
CARTWSGSYLSWFDPW	3-30-3	5	1	1	0
CVRSYSGSYFSWFDPW	3-30	5	1	1	0
CTRYYSGSYYYGMDVW	3-30-3	6	1	1	0
CATTRGGSYYGAFDIW	3-30	3	1	1	0
CVRGVGGGYYYGMDVW	3-30-3	6	1	1	0
CATVPIFGVVSWFDPW	1-24	5	1	1	0
CERETGDSSSSYFDYW	3-30	4	1	1	0
CARVVGANYYYGMDVW	3-30	6	1	1	0
CASVSSGYYYGAFDYW	3-30	4	1	1	0
CARSVYGDYYYYMDVW	4-59	6	1	1	0
CARTYSGSYYTPFDYW	3-30-3	4	1	1	0
CSRVSVVYYYYGMDVW	3-48	6	1	1	0
CARESIVVVHDAFDIW	3-30	3	1	1	0
CARTYYYDSSGYWDYW	3-30	4	1	1	0
CATTYSGSYKGYFDYW	3-30	4	1	1	0
CATSPAALRSNWFDPW	1-24	5	1	1	0
CAKDPVGATYDALDIW	3-23	3	1	1	0
CARTYSGIYLAPFDYW	3-30	4	1	1	0
CARARGGYYSRPFDYW	3-30	4	1	1	0
CARSGSGSYFAYFDYW	3-30	4	1	1	0
CARGWSGSYRTYFDYW	3-30-3	4	1	1	0
CATGPVVVAATWFDPW	1-24	5	1	1	0
CSRPGSGSYLSWFDPW	3-30	5	1	1	0
CATSAVAGTWGWFDPW	1-24	5	1	1	0
CATSPAIAVAGWFDPW	1-24	5	1	1	0
CARHWDNFWSGYYHYW	5-51	4	1	1	0
CATGPVAGTSNWFDPW	1-24	5	1	1	0
CARVLSGSYWGWFDPW	3-30	5	1	1	0
CPRALWGNYYYGMDVW	3-30-3	6	1	1	0
CARSRGGSYSNAFDIW	3-30	3	1	1	0
CARTSRGSYYDAFDIW	3-30	3	1	1	0
CARGSSGTYYYGMDGW	3-30	6	1	1	0
CARVSYDSLTGYYDYW	3-13	4	1	1	0
CARTNIAAAGTAVDYW	2-70	4	1	1	0
CARTSGGSYRGWFDPW	3-30-3	5	1	1	0
CASSNSGSYWGAFDIW	3-30	3	1	1	0
CARDVGSGWYGYFDYW	3-33	4	1	1	0
CATGPTGSYYDYFDYW	3-30	4	1	1	0

CASPYSGSYYSHFDYW	3-30	4	1	1	0
CARSSRGSYLNWFDPW	3-30	5	1	1	0
CARELIAAAGYYFDYW	3-33	4	1	1	0
CARSRSGSYSSAFDYW	3-30	4	1	1	0
CATSEVAGPLNWFDPW	1-24	5	1	1	0
CPRGYSGSYYEYFQHW	3-30	1	1	1	0
CARVGGGSYFNWFDPW	3-30-3	5	1	1	0
CASPRGGSYYGEFDYW	3-30-3	4	1	1	0
CARDLSGSYYYGLDVW	3-30-3	6	1	1	0
CARDSSGSYYGPNDYW	3-30-3	4	1	1	0
CARVYSGSYRGFFDYW	3-33	4	1	1	0
CARVTYGNYYYGMDVW	3-30	6	1	1	0
CATGPPFGVVSWFDPW	1-24	5	1	1	0
CARVHSGSYYGDFDYW	3-30-3	4	1	1	0
CARPYSGSYWSYVDYW	3-30-3	4	1	1	0
CARSYSGSYYHAFDIW	3-33	3	1	1	0
CAKTLSGSYYSPFYYW	3-30	4	1	1	0
CARPYSGSYFAQFDYW	3-30-3	4	1	1	0
CANPYSGSYRDAFDIW	3-30	3	1	1	0
CARAYSGSYYYGMDVW	3-53	6	1	1	0
CARGRGGGYHDAFDIW	3-30	3	1	1	0
CVTTTIFGVKGWFDPW	1-24	5	1	1	0
CARGTRGSYYYGMDVW	3-30-3	6	1	1	0
CARDLVGAAYYGMDVW	3-33	6	1	1	0
CARVTGANYYYGMDVW	3-30	6	1	1	0
CARSKGGGYYPPFDYW	3-30	4	1	1	0
CARTYSGSYYSYFDDW	3-30-3	4	1	1	0
CARVYSGSYRGHFDYW	3-30	4	1	1	0
CAKGKMGGYYYGMDVW	3-30	6	1	1	0
CARTNGGGYYYGMDVW	3-33	6	1	1	0
CASASGGNYYYGMDVW	3-30-3	6	1	1	0
CARGWSSGWSYYFDYW	3-53	4	1	1	0
CARGLRFLEWYYFDYW	4-34	4	1	1	0
CATASVFGVANWFDPW	1-24	5	1	1	0
CARSRSGSYTSWFDPW	3-30-3	5	1	1	0
CARVSSGSYRSAFDIW	3-30-3	3	1	1	0
CARGFGGSYYYGMDVW	3-53	6	1	1	0
CARSLGGSYYVAFDIW	3-30	3	1	1	0
CARTSGSYYYYFGMDVW	4-59	6	1	1	0
CARDTYYDILTGYYLYW	3-21	4	1	1	0
CARGVDLYYYYYGMDVW	4-34	6	1	1	0
CARSGYYYDSSGYSDYW	3-21	4	1	1	0
CARRYYGYYYYYGMDVW	4-39	6	1	1	0
CATPRYYYDSSGTFDYW	3-23	4	1	1	0
CAKFADYYDSSGYYDYW	3-9	4	1	1	0
CARDTNYDILTGYYSYW	3-21	4	1	1	0
CAREGQIAVAGTGLDYW	3-33	4	1	1	0
CATSPPMAAAGNWFDPW	1-24	5	1	1	0
CARDVDASGYYYGMDVW	3-7	6	1	1	0
CATGGGYYYYYYGMDVW	3-30	6	1	1	0
CARDLAVAGTSEYFQHW	3-33	1	1	1	0
CAREVIAVAGSNWFDPW	3-30	5	1	1	0
CARESLYGDYGGAFDIW	3-33	3	1	1	0

CARAGGSGSYYGWFDPW	3-30-3	5	1	1	0
CARVGRTIFGVVTNFDYW	4-31	4	1	1	0
CARRGYYDSSGYYYFDYW	5-51	4	1	1	0
CAREYYYDSSGYYPRDYW	1-18	4	1	1	0
CATSPSIAAAGNNWFDPW	1-24	5	1	1	0
CSKDYDSSGYYSDAFDIW	3-23	3	1	1	0
CATGPYCGGDCYGAFDIW	1-69	3	1	1	0
CARSNYDFWSGYLPFDYW	3-7	4	1	1	0
CVRNYDILTGYSDAFDIW	3-53	3	1	1	0
CAREGYSSSWSLYYFDYW	3-33	4	1	1	0
CARDVFYDFWSGYYFDYW	1-3	4	1	1	0
CAKNGYSYGYGGYYFDYW	3-30	4	1	1	0
CAKDSNGSGSYHDAFDIW	3-23	3	1	1	0
CARASYDSSGYYAYFDYW	3-13	4	1	1	0
CAREVDIVVVPAAPFDYW	1-46	4	1	1	0
CASGAVAGYYYYYGMDVW	3-21	6	1	1	0
CARDDYDYVWGSYSTDYW	3-30	4	1	1	0
CAKDRYYYDSSGYYYFDYW	3-23	4	1	1	0
CARSYYDILTGYLAAFDIW	2-5	3	1	1	0
CARIPYDILTGSLYGMDVW	2-70	6	1	1	0
CATIAAAGTHYYYYGMDVW	3-48	6	1	1	0
CASDYYDSSGYYNDWFDPW	3-30	5	1	1	0
CTTDRLGATYYYYYGMDVW	3-15	6	1	1	0
CSRDYYDSSGYYNNWFDPC	3-30-3	5	1	1	0
CARTHYGGNYYYYYGMDVW	3-13	6	1	1	0
CARYCSSTSCYTDDAFDIW	4-39	3	1	1	0
CARGGYSYGWSYYYGMDVW	3-53	6	1	1	0
CARVKRITIFGVVTHFDYW	4-59	4	1	1	0
CARGYYDILTGYYREFDYW	2-70	4	1	1	0
CATNFAYCGGDCYSRFDYW	1-24	4	1	1	0
CARGRGIQLWNYYYGMDVW	4-31	6	1	1	0
CATTSPYCSSTSCPRWFDPW	1-24	5	1	1	0
CSRGRYSSSWYGVAEYFQHW	4-34	1	1	1	0
CATDLYCSGGSCYSFGMDVW	3-23	6	1	1	0
CATLAAAGPEWYYYYGMDVW	3-21	6	1	1	0
CVRRRYSSSWPDYYYGMDVW	5-51	6	1	1	0
CARSYCSSTSCYGYYYMDVW	4-59	6	1	1	0
CARGGLAVAGTYYYYGVDVW	3-21	6	1	1	0
CARDPDYYDSSGYYVGFDYW	3-30-3	4	1	1	0
CARLTTVTTNYYYYYGMDVW	1-69	6	1	1	0
CARGRYSSSWYGVRNWFDPW	4-31	5	1	1	0
CARVVGGYCSSTSCYYFDYW	1-18	4	1	1	0
CATVGVYCTNGVCSNWFDPW	1-24	5	1	1	0
CARDLTYYYDSSGWGHFDYW	3-30	4	1	1	0
CAREVYDSSGYYYEDWFDPW	3-74	5	1	1	0
CARGYWNYVRYYYYYGMDVW	1-69	6	1	1	0
CARGTTVTTLFYYYYGMDVW	4-39	6	1	1	0
CVRGIVVVPAAHYYYYGMDVW	1-18	6	1	1	0
CARHPDIAVAGYYYYYGMDVW	4-59	6	1	1	0
CARWGWGSGSPNYYYYYMDVW	1-69	6	1	1	0
CARSGAGYSYGYAYYYYMDVW	1-69	6	1	1	0
CARGRWFGELLSYYYYGMDVW	4-4	6	1	1	0
CARVGLGYCSSTSCYTAFDIW	4-39	3	1	1	0

CARESGGYCSGGSCPNWFDPW	1-46	5	1	1	0
CARGRWFGELLSYYYYGMDVW	4-31	6	1	1	0
CARVGVLRYFDWQAYYYGMDVW	4-39	6	1	1	0
CARGGVYYYDSSGYYLDAFDIW	1-2	3	1	1	0
CARGRIAAAGLCNYYYYGMDVW	4-34	6	1	1	0
CVRGRRITIFGVVIDSFGMDVW	4-34	6	1	1	0
CARVTVLRYFDWSYYYYGMDVW	4-31	6	1	1	0
CARDSRFWEWLLYSYYYYMDVW	3-21	6	1	1	0
CARVTYYYDSSGYPFYWYFDLW	3-13	2	1	1	0
CARNGLYYYDSSGYYLDAFDIW	3-23	3	1	1	0
CARTGVLRYFDWSYYYYGMDVW	4-61	6	1	1	0
CARVPLLRYFDWTYYYYGMDVW	4-59	6	1	1	0
CARVGVLRYFDWLLHYYGMDVW	4-39	6	1	1	0
CAKVVGPYCSGGSCYSGQLDYW	3-30	4	1	1	0
CARGGYGSGSYYNYYYYYMDVW	1-69	6	1	1	0
CARDSVLRYFDWLPYYYGMDVW	4-61	6	1	1	0
CARDSYSSSWLGSYYYYGMDVW	3-7	6	1	1	0
CARDRYCSSTSCYRDYYYGMDVW	1-18	6	1	1	0
CARGWIVVVPAATYYYYYGMDVW	4-34	6	1	1	0
CARDRYCSGGSCYVYYYYGMDVW	1-18	6	1	1	0
CARVSYSSSWYWGDYYYYGMDVW	1-69	6	1	1	0
CARDTYCSGGSCYYYYYYGMDVW	3-7	6	1	1	0
CARVWGAGYSSGWYYYYYYMDVW	4-59	6	1	1	0
CARDVSSSWYSSGWYYYYYMDVW	3-7	6	1	1	0
CAKMGGGPYCSGGSCYSNYFDYW	3-30	4	1	1	0
CARGYTMVRGVIPRYYYYYMDVW	3-13	6	1	1	0
CARVGYCSSTSCYVPYYYYGMDVW	1-2	6	1	1	0
CARDVDIVVVPAATNYYYYGMDVW	3-21	6	1	1	0
CARDVTYYDILTGYYPQYYYYGMDVW	3-33	6	1	1	0
CARHLYYYDSSGYYYPPYYYYGMDVW	3-30	6	1	1	0
CARYQYYYDSSGYYYPTPYYYGMDVW	3-30-3	6	1	1	0
CARVGGGMDVW	1-46	6	1	0	1
CVKERQWLAYFDYW	3-30	4	1	0	1
CARGRYYDSSGLDYW	3-74	4	1	0	1
CARELVAGYYGMDVW	3-30	6	1	0	1
CARDSYSSSWYFDYW	3-30	4	1	0	1
CARGRGSYYGWFDPW	3-30	5	1	0	1
CARGYSSGYYYYFDYW	3-13	4	1	0	1
CARDPSSSWYIYFDYW	3-30	4	1	0	1
CASDPYYYDSSGLLDYW	3-21	4	1	0	1
CARSYDILTGYLYFDYW	3-30	4	1	0	1
CARVRYYDSSGYYSPFDYW	1-69	4	1	0	1
CARDLAVAATYYYYYGMDVW	3-7	6	1	0	1
CARDSGVAAAGNYYYYGMDVW	3-7	6	1	0	1
